# Fecal microbiota transplantation from warthog to pig confirms the influence of the gut microbiota on African swine fever susceptibility

**DOI:** 10.1038/s41598-020-74651-3

**Published:** 2020-10-19

**Authors:** Jinya Zhang, Fernando Rodríguez, Maria Jesus Navas, Mar Costa-Hurtado, Vanessa Almagro, Laia Bosch-Camós, Elisabeth López, Raul Cuadrado, Francesc Accensi, Sonia Pina-Pedrero, Jorge Martínez, Florencia Correa-Fiz

**Affiliations:** 1 IRTA, Centre de Recerca en Sanitat Animal (CReSA, IRTA), Campus de la Universitat Autònoma de Barcelona, Bellaterra, 08193 Barcelona, Spain; 2 OIE Collaborating Centre for the Research and Control of Emerging and Re-emerging Swine Diseases in Europe (IRTA-CReSA), Bellaterra, Barcelona, Spain; 3 Veterinary Service Zoo Barcelona, Parc Ciudadella s/n 08003, Barcelona, Spain; 4 Departament de Sanitat i Anatomia Animals, Universitat Autònoma de Barcelona, Bellaterra, 08193 Barcelona, Spain

**Keywords:** Microbial communities, Pathogens, Virology, Immunology, Molecular biology

## Abstract

African swine fever virus (ASFV) is the causative agent of a devastating hemorrhagic disease (ASF) that affects both domestic pigs and wild boars. Conversely, ASFV circulates in a subclinical manner in African wild pigs, including warthogs, the natural reservoir for ASFV. Together with genetic differences, other factors might be involved in the differential susceptibility to ASF observed among Eurasian suids (*Sus scrofa*) and African warthogs (*Phacochoerus africanus*). Preliminary evidence obtained in our laboratory and others, seems to confirm the effect that environmental factors might have on ASF infection. Thus, domestic pigs raised in specific pathogen-free (SPF) facilities were extremely susceptible to highly attenuated ASFV strains that were innocuous to genetically identical domestic pigs grown on conventional farms. Since gut microbiota plays important roles in maintaining intestinal homeostasis, regulating immune system maturation and the functionality of the innate/adaptive immune responses, we decided to examine whether warthog fecal microbiota transplantation (FMT) to domestic pigs affects host susceptibility to ASFV. The present work demonstrates that warthog FMT is not harmful for domestic weaned piglets, while it modifies their gut microbiota; and that FMT from warthogs to pigs confers partial protection against attenuated ASFV strains. Future work is needed to elucidate the protective mechanisms exerted by warthog FMT.

## Introduction

African Swine Fever (ASF) is a devastating disease of domestic pigs and wild boars, caused by African swine fever virus (ASFV). ASF is a notifiable disease to the World Organization for Animal health (OIE)^1^ and today it is considered the most serious constraint for pig production. The current distribution of African swine fever extends across more than 50 countries from African, Asian and European continents and more recently, also from Oceania^2^.

ASFV is a large enveloped virus of approximately 260 to 300 nm in diameter^3^ with a genome size between 170 and 193 kbp^4^ encoding at least 150 different proteins^5^ and the only known DNA arbovirus. ASF was described for the first time in 1921 as a new disease affecting domestic pigs in Kenya^6^. Before domestic pigs were introduced into Africa, ASFV was circulating following a sylvatic cycle between soft ticks (*Ornithodoros)* and African warthogs (*Phacochoerus africanus*). Warthogs and bushpigs (*Potamochoerus porcus*) act as ASFV reservoirs in the wild^7^. Depending on the viral isolate, domestic pigs infected with ASFV can develop a disease that ranges from chronic or subclinical to subacute and hyper-acute^8^, resulting the latter in up to 100% mortality in naïve pigs^9^. The mechanisms of ASF-resistance showed by warthogs and bush pigs has not yet been elucidated, albeit both genetic^6,10^ and environmental factors could be involved. Preliminary experimental evidences described local pigs in Africa as less susceptible to infection with certain ASFV genotypes^10^. On this regard, it is worth to mention that specific-pathogen-free (SPF)-pigs were more susceptible to infection with ASFV attenuated strains than genetically identical pigs raised in conventional farms^11^, allowing to hypothesize that, together with genetics, warthog microbiota could contribute to ASF-resistance.

The intestinal microbiota affects multiple facets of organism homeostasis through its influence on the innate immune system^12,13^. The gut microbiota of the animal species mentioned above, i.e. warthogs and both SPF- and domestic pigs, have been recently unveiled, showing relevant differences in composition^14^. Fecal microbiota transplantation (FMT) is a delivery of donor microbiome to a recipient in order to establish or restore intestinal homeostasis, or populate the gastrointestinal tract with potentially beneficial bacteria^15^. Interest on the novel FMT for the prevention and treatment of intestinal disorders has been increasing in human medicine, for example, to control *Clostridium difficile* infection^16^ or inflammatory bowel disease^17^. More and more clinical applications of FMT have provided convincing proofs that modification of the intestinal microbiota is an effective therapy for intestinal dysbiosis-related diseases^18^. In pigs, published studies using FMT are scarce, but they provide evidence of the ability to reprogram the porcine intestinal microbiota via FMT, altering immune phenotype of the host^19^.

In the present work, weaned piglets were first, transplanted with fecal microbiota from either warthogs or domestic pigs, and second, they were challenged with a virulent or an attenuated ASFV strain to compare their disease outcomes. No differences were observed after intramuscular challenging with E75, a virulent ASFV strain, independently of the FMT. Conversely, a very significant reduction of virus in serum, nasal viral shedding and clinical signs were observed when pigs transplanted with warthog feces were intramuscularly challenged with E75CV1, an attenuated ASFV strain, when compared with pigs transplanted with domestic pig feces. Far from understanding the mechanisms involved in the protection afforded, we provide here evidences showing the protective potential to ASF of warthog microbiota.

## Results

### FMT modifies the gut microbiota diversity in transplanted pigs

Forty-eight 21-day-old animals divided into four groups were orally inoculated over three consecutive days with either a pool of warthog feces (WF group), a pool of domestic pig feces (PF group) or PBS (control group). The fourth group of pigs was treated with a cocktail of antibiotics one day before inoculation with a pool of warthog feces (AWF group), aiming to facilitate the warthog microbiota transplantation. Fecal samples from 5 pigs from each group (PF, WF, AWF and PBS) were collected at 15 days post fecal transplantation (dpft) and their fecal microbiota was compared. The 16S ribosomal DNA (rRNA gene) was sequenced individually from all these samples. After quality trimming processes, 730,952 high-quality sequences were obtained for 20 feces samples. The read count for each sample ranged from 1425 to 60,027, with a mean frequency of 36,548. Since the read count from one animal (PF#13) was too low (1425) in comparison with the mean frequency, it was discarded from the analysis (Supplementary Table S1).

**Figure 1:**
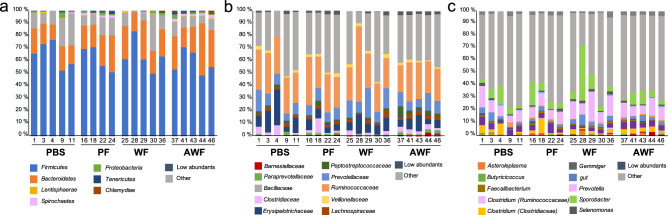
Fecal microbiota composition at 15 days post-fecal-transplantation (dpft) in WF, AWF, PF and PBS groups. Each bar represents the relative abundance of taxa found in feces at different taxonomical levels: phylum (**a**), family (**b**), genus (**c**). The taxa found with less than 1% of relative abundance were collapsed as ‘low abundants’. The ten most relatively abundant taxa are shown in the legend. For a full list of the taxa composing the fecal microbiota composition, please refer to Supplementary Table S2.

The observed taxa at different taxonomic levels for different groups are shown in Fig. 1. The classified taxa were distributed in 15 phyla, 60 families, and 77 genera. *Firmicutes* and *Bacteroidetes* were the two dominant phyla for all the groups, followed by *Spirochaetes* or *Proteobacteria*. In relative terms, the most abundant family in all the groups was *Ruminococcaceae*, while the second most abundant family was *Prevotellaceae* for all transplanted groups (PF, WF and AWF) and *Erysipelotrichaceae* for PBS. *Prevotella* was the dominating genus in PBS, WF and AWF while *Sporobacter* was dominant for PF group (Supplementary Table S2).

**Figure 2:**
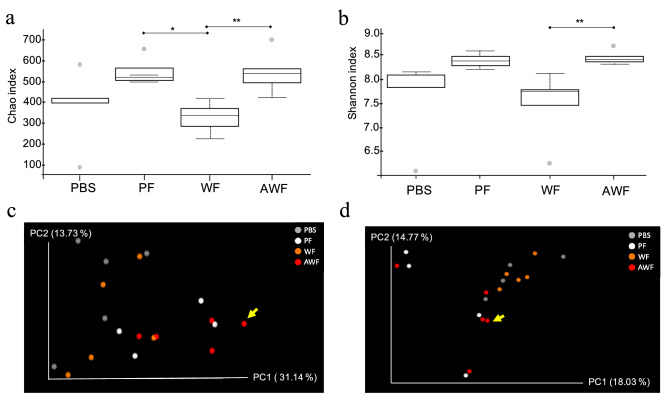
Alpha and beta diversity on rarefied fecal samples at 15 days post-fecal-transplantation (dpft) in WF, AWF, PF and PBS groups. Alpha diversity computed through Chao1 index (**a**) or Shannon–Wiener’s metrics (**b**). Dotted lines represent the standard deviation and outliers are indicated with grey circles. *P < 0.05 and **P < 0.01. Beta diversity was calculated through weighted (**c**) and unweighted (**d**) Unifrac distances at 15dpft. The principal axes are shown with the percentage of variation explained between brackets. Arrows indicate the microbiota composition for animal #44.

Alpha diversity was estimated for all the groups at 15 dpft through Chao1 index that considers richness (Fig. 2a) and Shannon index that considers both richness and evenness (Fig. 2b). The richness (Fig. 2a), showed a tendency to be higher in both PF and AWF in comparison with PBS (*P* = 0.08 and *P* = 0.07 respectively), while proved to be statistically higher than WF (*P* = 0.01 and *P* = 0.009 respectively). This increase in richness was accompanied with an increase in diversity, only for AWF (Fig. 2b), showing a Shannon index statistically higher than WF (*P* = 0.009) and a tendency to be higher than PBS (*P* = 0.07).

The spatial changes in bacterial communities among groups was explored through PCoA. Beta diversity analysis was done using weighted (Fig. 2c) or unweighted Unifrac phylogenetic (Fig. 2d) distances. PCoA was performed to visualize the differences in Unifrac distances for all samples at 15 dpft. The beta diversity analysis showed a distinct clustering comparing the different groups, explaining 28.44% of the differences in the quantitative analysis (weighted, *P* = 0.007) and 21.87% in the qualitative analysis (unweighted, *P* = 0.021). Interestingly, pig AWF#44 showed a dissimilar microbiota composition (Fig. 2b) with the highest mean distance (0.241 ± 0.02) compared to the mean of the AWF group (0.199 ± 0.03).

### Warthog fecal microbiota transplantation is not harmful

Transplantation was done using weaned piglets (21 days-old) and clinical parameters were recorded for 30 days. Despite the delicate transition suffered during weaning, warthog fecal transplantation did not harm the animals. The diarrhea observed in the AWF group lasted for 2–3 days, starting with the antibiotic treatment and finishing the third and last day of FMT.

**Figure 3:**
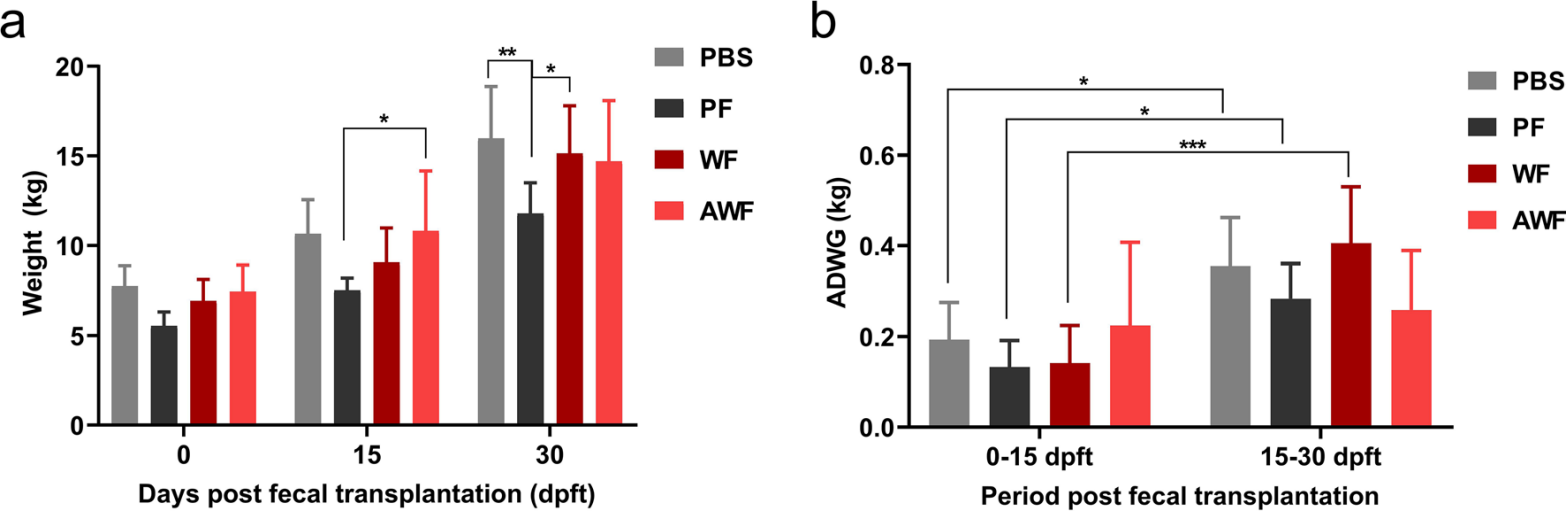
Warthog FMT do not harm the domestic pigs in terms of weight and average daily weight gain (ADWG). Comparison of average weight at 0, 15 and 30 dpft (**a**) and ADWG calculated for two periods: 0 to 15 dpft and 15 to 30 dpft in PBS, PF, WF and AWF groups. *P < 0.05, **P < 0.01 and ***P < 0.005.

No significant differences were observed between pigs transplanted with warthog feces (WF and AWF) and the PBS group in terms of mean weight or average daily weight gain (ADWG) from 0 to 30 dpft (Fig. 3a,b). The mean weight of PF group was lower than the AWF group at 15 dpft and was the lowest from all other groups at 30 dpft (Fig. 3a). The ADWG increased significantly in time for WF, PBS and PF groups when compared the first and the second fortnights (Fig. 3b). While the AWF group showed the same trend of weight gain during the period, WF group showed different growth dynamics since the animals within this group gained less weight during the first 15 days, but their growth improved dramatically in the second fortnight. On the other hand, PF pigs gained less weight than PBS control group during the whole observation period (Fig. 3b), a group that showed diarrhea, coinciding also with the shortest colon crypt depths among all the groups (Supplementary Table S3).

**Figure 4:**
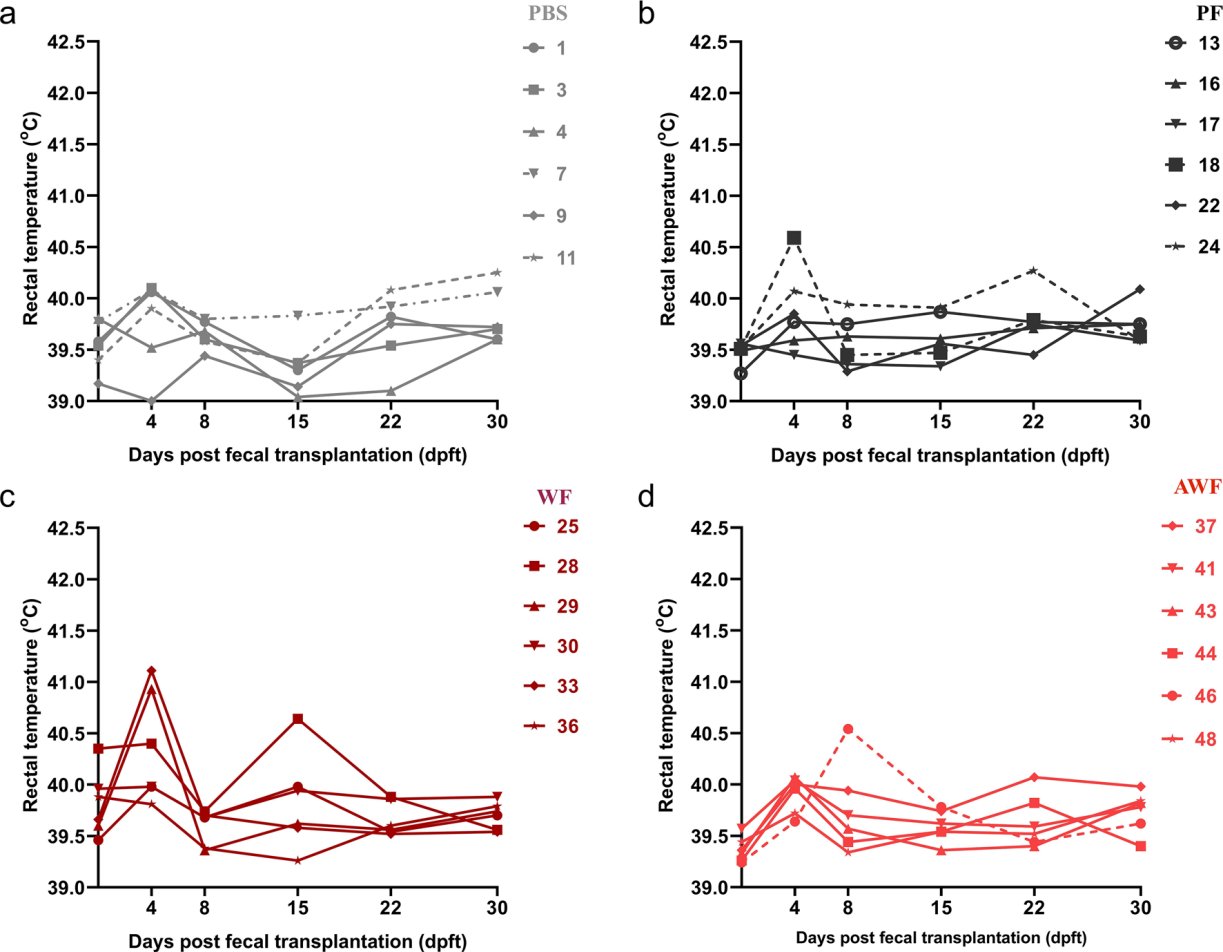
Comparison of rectal temperatures between groups at different days post-fecal transplantation (dpft). Rectal temperature was taken on 0, 4, 8, 15, 22 and 30 dpft for PBS (**a**); PF (**b**); WF (**c**); and AWF groups (**d**). Transplanted pigs (PF, WF and AWF) showed constant rectal temperature in the late period after FMT.

Some transplanted pigs (PF#13, PF#18, WF#28, WF#29, WF#33 and AWF#46), showed a brief increase in the rectal temperature (RT) in the period from 4 to 8 dpft (Fig. 4b,c,d), not observed in PBS animals (Fig. 4a). Conversely, from 15 to 30 dpft, RT remained normal and constant in WF, AWF and PF pigs, while PBS pigs’ RT showed evident oscillations.

### Warthog fecal microbiota transplantation confers partial protection against ASF in vivo

**Figure 5:**
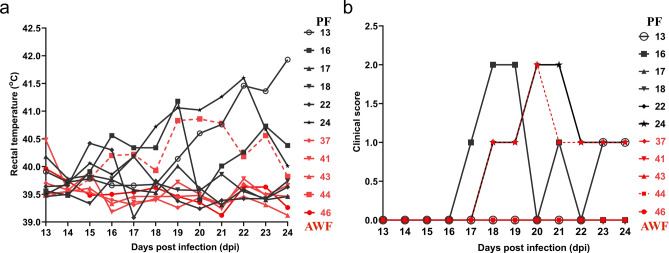
Comparison of rectal temperatures (**a**) and chronic ASF-compatible clinical signs (**b**) from PF (in black) and AWF (in red) groups after infection with attenuated E75CV1 strain. Clinical scores were calculated considering body condition, behavior, digestive respiratory and other significant clinical signs including arthritis, dermatitis, testicular tumefaction.

To evaluate the effects of warthog FMT on ASF susceptibility, transplanted domestic pigs were infected with either the E75 virulent strain or the E75CV1 cell culture-adapted strain^20^. Thirty days after FMT, the animals were transported to BSL3 facilities. PF and AWF were intramuscularly challenged with 100 hemagglutinin units (HAU) of the attenuated E75CV1 strain, facilitating the observation of potential anti-viral effects of the warthog microbiota; while PBS and WF pigs were infected with 10^4^ HAU of the parental virulent E75 virus, a more severe ASFV acute lethal challenge. Animals were observed daily according to a welfare schedule to monitor their health status and to record the clinical signs after the infection of ASFV (Supplementary Table S4). Correlating with previous observations, after intramuscular inoculation of 100 HAU of E75CV1^20^, 50% of the PF (#13, #16, #24) showed consistent elevated rectal temperatures late after infection (Fig. 5a), starting at 15 dpi (days post infection) and prolonged until day 23 dpi, with only PF#13 showing fever by the end of the experiment (24 dpi). Conversely, only AWF#44, showed fever comparable to that observed for PF pigs (Fig. 5a). Mild clinical signs compatible with chronic ASF were observed (Fig. 5b), perfectly matching with the fever profile observed, with pigs PF#13, PF#16, PF#24 and AWF#44, showing testicular and joint inflammation late after infection. With the exception of AWF#48 that accidentally died while blood sampling at 13 dpi, no other significant clinical findings were recorded in AWF group. At 24 dpi, all animals were euthanized but no gross or microscopic lesions were observed in any animal.

**Figure 6:**
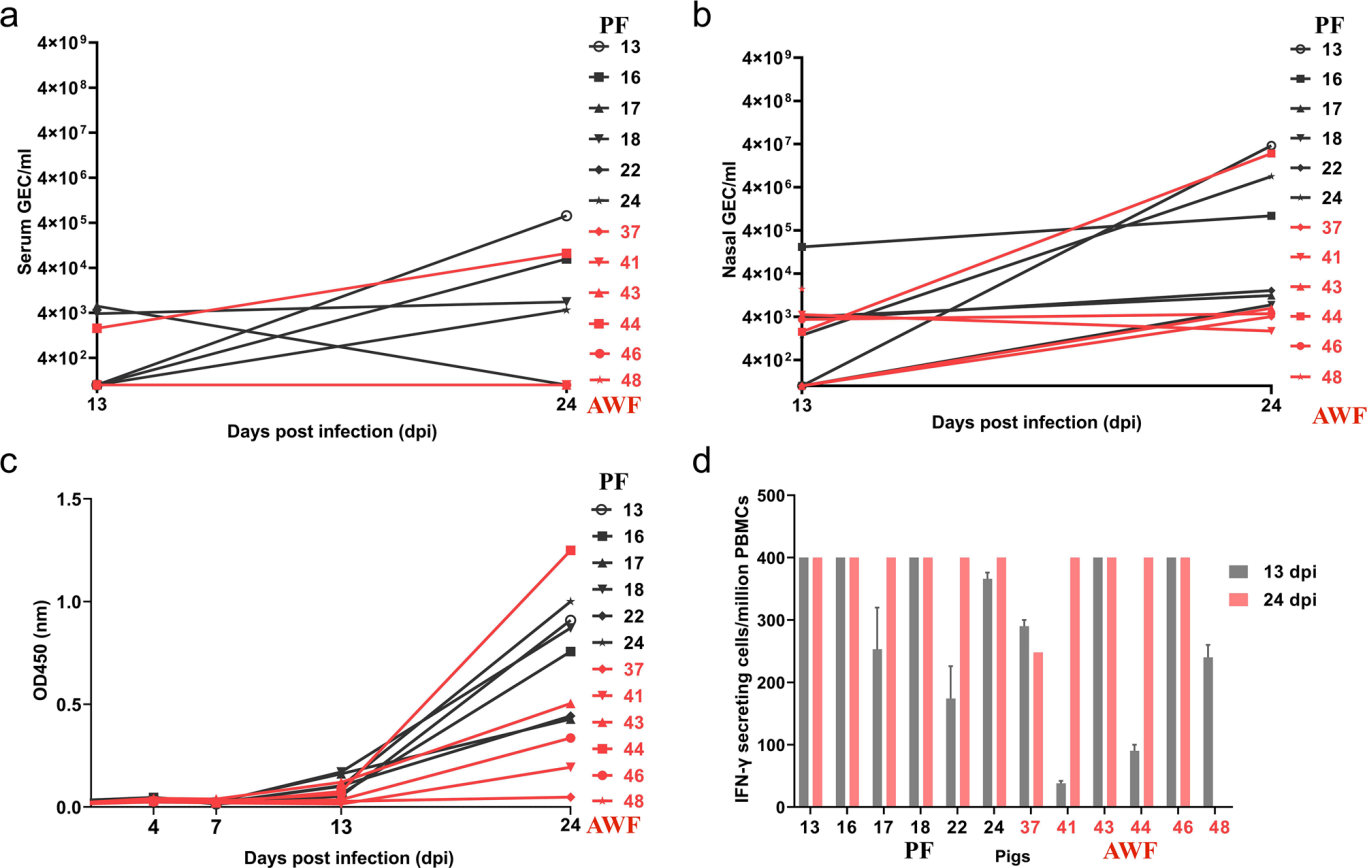
Comparison of virus titration in serum (**a**), nasal viral excretion (**b**), ASFV-specific antibodies production detected by ELISA (**c**) and specific T-cell responses measured in an IFNγ-ELISPOT (**d**) between PF (in black) and AWF (in red) groups after infection with attenuated E75CV1 strain. The limit of detection of the qPCR is 1 gene equivalent copies (GEC) of ASFV genome/µl of serum or nasal swab homogenate, while the maximum number of positive spots quantifiable in the ELISPOT is 400 spots/million PBMCs.

Additionally, the presence of significant virus in serum (Fig. 6a) and viral shedding (Fig. 6b) were also high for PF#13, PF#16, PF#24 and AWF#44, and paralleled with fever and clinical signs in the infected pigs (Fig. 5). PF and AWF pigs, independent of the origin of the transplantation material, mounted detectable cellular and humoral responses against ASFV. The amount of ASFV specific antibodies found in the serum of E75CV1 infected pigs seemed to correlate with the virus titers found in serum and nasal swabs, with PF#13, PF#16, PF#24 and AWF#44 showing the highest antibody titers by day 24 dpi (Fig. 6c). No differences were observed for the ASFV-specific T-cell responses, independent of the fecal microbiota origin. ASFV-specific T-cells were detectable in all pigs by IFNγ-ELISPOT as early as at 13 dpi and remained present until the end of the experiment (Fig. 6d).

As above-mentioned, the protection failure observed in pig AWF#44, seemed to correlate with a different microbiota composition at 15 dpft than the rest of the pigs (Fig. 2c,d). Confirming this observation, the *Firmicutes*/*Bacteroidetes* ratio, commonly used as a health indicator^21–24^, gave a value of 1.16 for the AWF#44 at 15 dpft, much lower than the rest of the pigs within the AWF group that showed an average ratio of 2.323 (Table 1).

**Table 1:** *Firmicutes/Bacteroides* ratio along time in PF and AWF groups after FMT.

TIme	PF					AWF				
	#13	#16	#18	#22	#24	#37	#41	#43	#44	#46
0 dpft	3.333	2.742	1.429	0.654	1.674	1.449	1.428	1.774	1.693	1.219
8 dpft	2.130	2.029	3.372	1.496	1.000	2.076	1.137	1.192	0.838	1.439
15 dpft	NA	3.718	3.962	2.251	1.739	2.003	4.502	3.235	1.160	1.875

*NA* not available (sample excluded from analysis), *PF* pig feces group, *AWF* antibiotic-treated warthog feces group, *dpft* days post-fecal transplantation.

**Figure 7:**
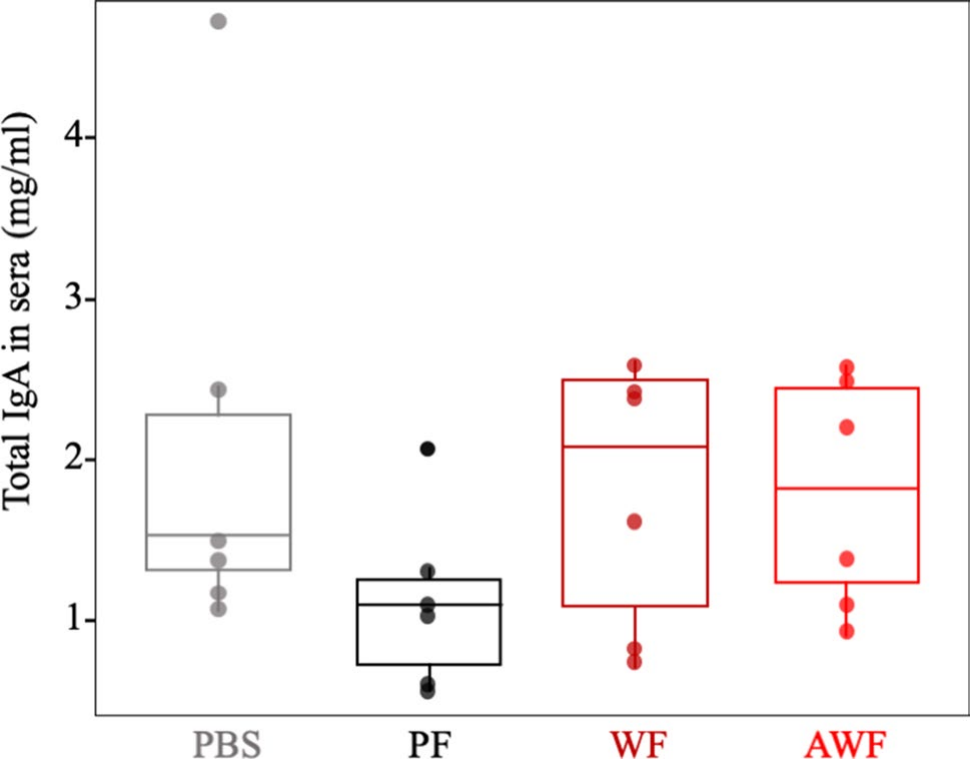
Effect of the FMT on the mucosal immunity. An increased tendence of IgA levels was found in pigs transplanted with warthog feces (WF and AWF) when compared with pig-feces transplanted animals (PF) or non-transplanted control pigs (PBS). No statistically difference was found between groups (Kruskal Wallis, *P* = 0.3). Plots were generated using ggplot2 package^25^ in R Studio software^26^ (Version 1.2.5033).

Despite the absence of direct and solid in-vitro correlates for ASFV protection, once more confirmed in here, we found that warthog FMT might enhance mucosal immunity. Thus, the total IgA found in sera from pigs transplanted with warthog feces (WF and AWF groups), showed a tendency to increase when compared with that found in PF and PBS groups (Fig. 7). The amount of ASFV-specific IgA found in the sera of E75CV1-infected pigs parallel that observed for the IgG and with the virus titers found in serum and nasal swabs.

**Figure 8:**
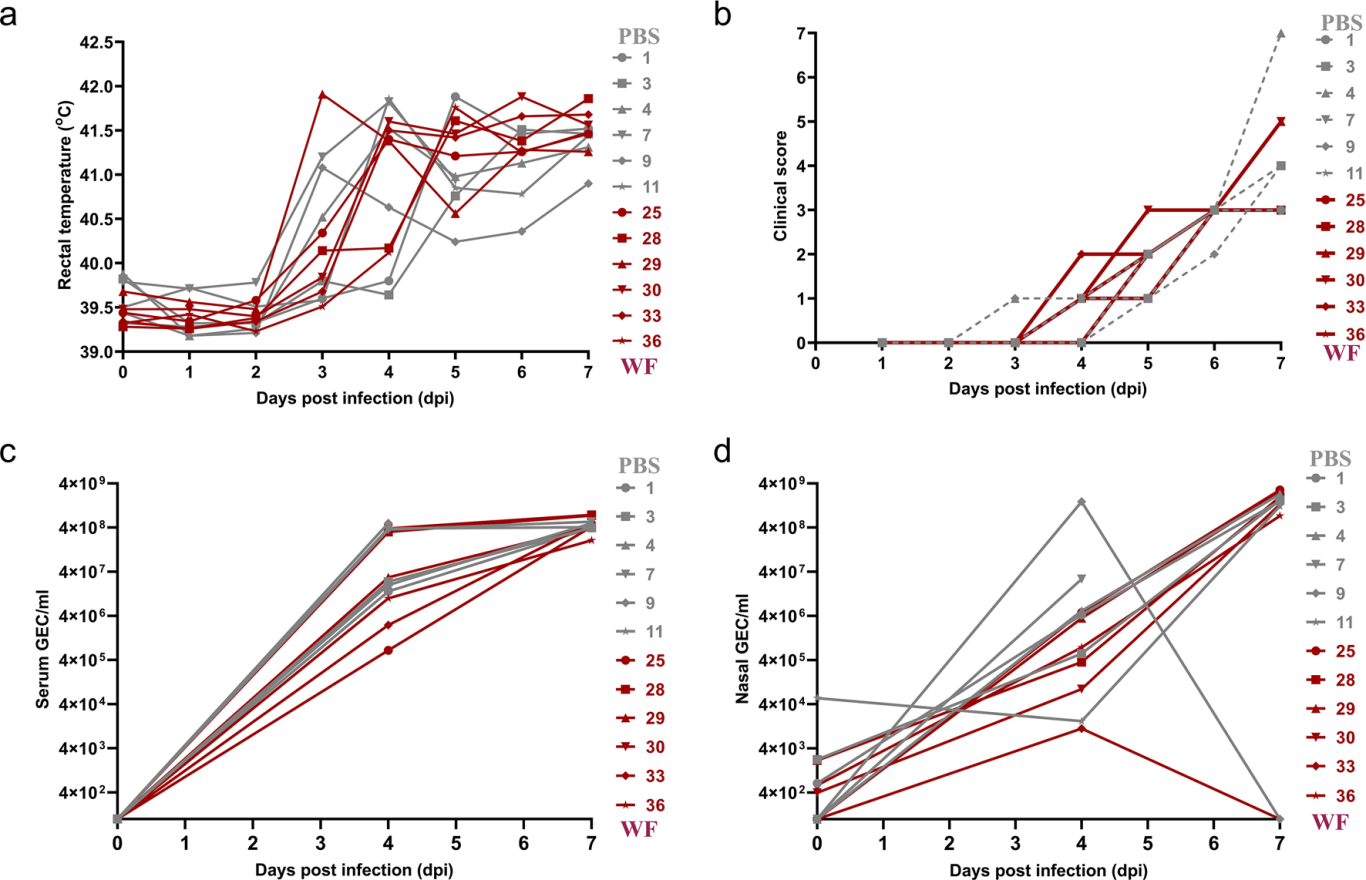
Comparison of rectal temperatures (**a**), clinical signs compatible with acute disease (**b**), virus titration in serum (**c**) and nasal swabs (**d**) between PBS (in grey) and WF (in dark red) groups after infection with virulent E75 strain. Clinical scores were calculated following a guide previously published by the group^27^.

In order to evaluate more carefully the anti-viral potency of warthog fecal microbiota, two additional groups of pigs (PBS and WF) were inoculated with a lethal dose of 10^4^ HAU of the E75 strain. As expected from our previous study^20^, PBS pigs developed acute clinical signs of ASF from 4 dpi (Fig. 8a,b), including anorexia, depression, redness and petechiae in the skin and high rectal temperature. WF pigs had clinical signs identical to those described for the PBS pigs (Fig. 8a,c). PBS pigs and WF pigs were humanely euthanized at 7 dpi. At necropsy, pigs showed similar lesions among groups consisting of multiple hemorrhages on serosal surfaces, mild ascites, interstitial edema of the lung and the mesentery, moderate to marked splenomegaly and hemorrhages in the gastro-hepatic lymph node. There was no difference in virus replication rate in both serum and nasal swabs (Fig. 8c,d).

### Longitudinal characterization of the PF and AWF microbiota composition

**Figure 9:**
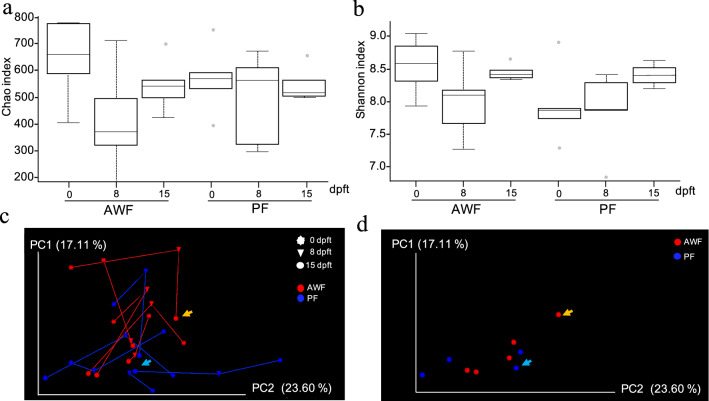
Longitudinal analysis on rarefied fecal samples from AWF and PF groups. Alpha diversity was computed through Chao1 index (**a**) or Shannon–Wiener’s metrics (**b**) at 0, 8 and 15 days post-fecal-transplantation (dpft). Dotted lines represent the standard deviation and outliers are indicated with grey points. Beta diversity was estimated through weighted Unifrac distances comparing AWF and PF at 0, 8 and 15 dpft (**c**). The microbiota composition of each animal at each timepoint is represented with triangles for 0 dpft, spheres for 8 dpft and hexagons for 15 dpft. The lines join the microbial composition for each animal at different timepoints (in red for AWF and blue for PF). The beta diversity for AWF and PF at 15 dpft is represented by disabling the visibility of other timepoints (0 and 8 dpft) for better visualization (**d**). The arrows indicate the microbial composition of AWF#44 and PF#22 at 15 dpft in orange and light blue, respectively. The principal axes are shown with the percentage of variation explained between brackets. *P < 0.05 and **P < 0.01.

Based on the results above described, we decided to complete the characterization of the fecal microbiota changes observed along time in both the AWF (E75CV1-resistant) and PF (E75CV1-sensitive) groups. Since the 16S rDNA from AWF and PF fecal samples was already characterized at 15 dpft (Figs. 1 and 2), additional samples from the same animals from these two groups (AWF and PF), collected before FMT (0 dpft) and at 8 days post-fecal-transplantation (8 dpft), were subjected to 16S ribosomal DNA sequencing and analysis. After quality trimming processes, 808,856 high-quality sequences were obtained for these 20 feces samples (Supplementary Table S1). The longitudinal microbiota analysis of fecal samples obtained at 0, 8 and 15 dpft from AWF and PF, showed that AWF richness, decreased between 0 and 8 dpft (*P* = 0.08), and recovered between 8 and 15 dpft (Fig. 9a). A similar pattern was observed regarding AWF diversity (Fig. 9b), when comparing 0 and 8 dpft (*P* = 0.1), showing also recovery between 8 and 15 dpft (*P* = 0.1). PF diversity showed a tendency to increase over time, although it was not statistically significant (0 to 8 dpft, *P* = 0.6; and 8 to 15 dpft, *P* = 0.08). The ANCOM (quantitative) analysis comparing the microbiota composition of AWF and PF at 15 dpft, identified four differential taxa present in AWF: *Mycoplasma* and *Chlamydia*, together with one unidentified genus from *Enterobacteraceae* family and another from the *Proteobacteria* phylum. No quantitative differences were found through ANCOM, either at 0 or 8 dpft.

Beta diversity analysis using weighted Unifrac distances, additionally demonstrated that the microbiota composition changed along the time (0, 8 and 15 dpft, Fig. 9c,d). Thus, the microbiota composition of AWF at 0 dpft was different from the composition at 8 dpft (PERMANOVA, *P* = 0.05) and at 15 dpft (*P* = 0.01). The differences for PF group only became evident when comparing 0 and 15 dpft (PERMANOVA, *P* = 0.02).

Finally, in an attempt to find qualitatively different taxa among groups we determined first, the core taxa found in AWF and PF at different times post-transplantation: 0, 8 and 15dpft and compared with the taxa composition of the warthog FMT inoculum (pWF) and the porcine FMT (pPF) inoculum used for the transplantation (Supplementary Table S1). As depicted in Table 2, six genera were present in pWF but absent pPF: *Desulfovibrio Roseburia, Ruminococcus, Actinobacillus, Faecalibacterium, Butyricicoccus*. After fecal transplantation, only *Faecalibacterium* was detected in AWF core at 8 and 15 dpft, while *Roseburia* and *Desulfovibrio* were detected only at 15 dpft. Four genera were detected only in pPF i.e. *Asteroleplasma, Paraprevotella Gemmiger* and *Treponema*. With the exception of *Gemminger*, all these genera were found in both transplanted groups at 15 dpft with similar relative abundance but *Treponema,* that was found in lower relative abundance in the AWF group. When we compared the core composition of the two transplanted groups at 15 dpft, we found four genera as exclusive members of the PF core microbiota: *Campylobacter, Clostridium* (from *Lachnospiraceae* family),* Actinobacillus* and *Succinispira*. While *Barnesiella*, *Desulfovibrio*, *Anaerorhabdus* and *Roseburia,* were part of the AWF core at 15 dpft. Finally, *Barnesiella* and *Anaerorhabdus,* they seemed to preferentially colonize pigs in the context of pWF FMT, since they were found exclusively in the AWF core at 15 dpft*,* although they were present with the same relative abundance in both the pPF and the pWF original inocula. Detailed information about the core taxa are shown in Supplementary Table S5.

**Table 2:** Mean relative abundance of core genera from the inoculum used and the different groups at different timepoints after FMT.

Taxa		Inoculum		PF			AWF		
Family	Genus	pPF	pWF	0 dpft	8 dpft	15 dpft	0 dpft	8 dpft	15 dpft
*Barnesiellaceae*	*Barnesiella*	1.114	0.772	4.018	1.717		2.347	3.06	1.217
*Campylobacteraceae*	*Campylobacter*	0.05	0.021			0.15			
*Clostridiaceae*	*Clostridium*	0.823	1.38	4.499		3.969	3.641		2.943
*Desulfovibrionaceae*	*Desulfovibrio*		0.12	0.177					0.065
*Erysipelotrichaceae*	*Unclassified*	0.976	0.741		0.821	2.161	0.945	1.211	2.428
*Erysipelotrichaceae*	*Asteroleplasma*	0.371				1.096		0.302	0.893
*Erysipelotrichaceae*	*Anaerorhabdus*	0.438	0.421		0.483		0.253		0.241
*Lachnospiraceae*	*Clostridium*	0.248	2.841			0.227		0.369	
*Lachnospiraceae*	*Defluviitalea*	0.155	0.082				0.094		
*Lachnospiraceae*	*Roseburia*		0.958	0.248					0.539
*Lachnospiraceae*	*Ruminococcus*		0.415	0.482					
*Paraprevotellaceae*	*Paraprevotella*	1.961			2.263	0.923	1.267	2.949	1.023
*Pasteurellaceae*	*Actinobacillus*		0.023			0.078			
*Peptostreptococcaceae*	*Clostridium*	0.143	0.532				2.191		
*Prevotellaceae*	*Prevotella*	4.951	3.015	9.536	9.566	8.428	19.217	17.029	9.916
*Ruminococcaceae*	*Sporobacter*	2.617	1.25	2.48	4.346	7.694	2.783	5.897	6.109
*Ruminococcaceae*	*Clostridium*	4.247	8.514	4.111	5.255	4.127	3.036	5.178	4.275
*Ruminococcaceae*	*Papillibacter*	0.222	0.273	0.285	0.321	0.217	0.378		0.224
*Ruminococcaceae*	*Faecalibacterium*		0.303			1.303		1.147	0.791
*Ruminococcaceae*	*Butyricicoccus*		0.994	0.358		0.51	0.414		
*Ruminococcaceae*	*Gemmiger*	0.248						0.546	
*Ruminococcaceae*	*Bacteroides*	0.203	0.192	0.219					
*Spirochaetaceae*	*Treponema*	1.24				0.481			0.217
*Veillonellaceae*	*Selenomonas*					1.283			0.869
*Veillonellaceae*	*Succinispira*	0.529	0.462	0.575		0.485	0.349	0.37	

## Discussion

Fecal microbiota transplantation has proven beneficial in the treatment of human and animal viral diseases^28^, chronic liver diseases^29^, ulcerative colitis^30^ and mainly, to fight multi resistant *Clostridium difficile* infections in humans^31,32^. FMT has confirmed the critical role that gut microbiota plays in early weaned-piglets in conferring diarrhea resistance^28,33^, using not fully understood mechanisms^34,35^.

Feeding animals with feces from the same or different species^36^ has been a traditional fattening practice which vanished from industrial farming due to sanitary problems, especially when using feces from the same animal species. The diarrhea observed in the PF group might be due to the unwanted transplantation of enteric pathogens present in the transplanted material, despite being selected from healthy sows. *Clostridium perfingens* type B has been isolated from sick animals, albeit most probably, other enteric pathogens might be responsible of the diarrhea observed. The fact that WF and AWF groups did not show any morbidity when compared with PBS control pigs, seems to confirm the innocuous nature of warthog feces for pigs.

Weaning is one of the most delicate periods in the pig’s life, when many stressful events occur contributing to intestinal and immune system dysfunctions that result in impaired pig health, reduced growth and feed intake, particularly during the first week after weaning^37^. Early intervention with FMT improved the maturation of the immune system alleviating weaning stress and reduced morbidity and mortality associated to porcine circovirus type 2 (PCV-2)^38^. Compared with weaned pigs from PBS group, WF animals showed a significant increase in their daily weight gain between 15 and 30 dpft. The PF group, however, rendered poor results compared to the rest of the groups, most probably due to accidental transplantation of specific swine enteric pathogens, as mentioned above. The prior use of antibiotics makes it difficult to compare AWF with the rest of the groups. Conversely to PBS, the increase on ADWG of WF became evident from 15dpft and not at early times, concurring with the early and transient peak of fever. Coinciding with the early RT increase, transplanted pigs showed higher percentages of monocytes in their blood at 4 dpft than those found in the PBS group (Supplementary Table S6), inverting this relationship by 15 dpft, most probably reflecting an early and transient inflammatory reaction, already described in the literature after fecal transplantation^39,40^. Conversely, the uniform and constant temperatures found between 15 and 30 dpft in transplanted pigs, independently of the feces origin (domestic pigs or warthogs), might reflect a synchronization in their circadian rhythms, as described in humans^41^. In this regard, pigs and warthog microbiota share with humans several common intestinal microbiota components, such as *Enterobacter aerogenes*, a bacteria that is sensitive to the pineal and gastrointestinal melatonin hormone, precisely working in circadian rhythms of 24 h between the 26 °C and 40 °C^42^. As expected, PBS control group animals showed differences in their body temperatures, within the physiological range.

Despite the changes described herein, transplantation of warthog feces did not dramatically change the microbiota of the transplanted piglets, or at least, there is not any enrichment in the few taxa previously described as unique from the warthog species^14^. These results coincide with that described in many reports, dissociating beneficial effects of FMT from easily detectable changes in host microbiota^32^, allowing hypothesizing with a beneficial stimulation of the immune system. Comparing ASF clinical score, ASFV in serum and nasal shedding of pigs from PF and AWF groups, AWF controlled E75CV1 infection better than PF, except for AWF#44 that has an anomalous microbiota composition compared with the rest of the animals within the AWF group. However, animals from both groups showed similar humoral and cellular responses, at least measured by ELISA and IFNγ-ELISPOT, respectively. This result confirms, once more, these measures^43^, as bad protection correlates (unfortunately, no correlates of ASF protection have been described so far), pointing towards more subtle differences in the innate/adaptive immune responses induced between both groups. In this regard, work performed in the last decades have allowed identifying T-cell responses as crucial in ASFV protection^20^. In particular, a direct correlation seems to exist between protection, Th1-like responses and the induction of specific CD8^+^ T-cells (cytotoxic T-lymphocytes) against ASFV^43^. Experimental immunization with attenuated strains also shows that this inflammatory response comes together with the induction of regulatory T-cells that tightly controls any excessive inflammation and that this equilibrium dictates somehow the safety and the efficacy and long-term duration of the vaccine^20,44^. Interestingly, most of the bacteria specifically found in transplanted pigs at 15 dpft, just before ASFV challenge, have been associated with anti-inflammatory states. This is the case for *Faecalibacterium,* considered a constitutive marker of a healthy gut for human^45^ and is associated with anti-inflammatory properties^46^. Interestingly, *Faecalibacterium* was the core taxa for AWF pigs at 8 dpft and 15 dpft and the relative abundance of *Faecalibacterium* for AWF#44 was different compared with the rest pigs under the same treatment, perhaps contributing to its susceptibility to ASFV infection. *Roseburia*^47^ is another member of the AWF core taxa at 15dpft, a bacteria capable to produce short-chain fatty acids (SCFA)^48–50^, metabolic mediators balancing of inflammatory and anti-inflammatory T cell responses subsets and antimicrobial peptides (AMPs) production^51^. From the other genera found exclusively in the AWF core at 15 dpft, *Barnesiella*^52^, has been directly involved in the reduction of the pathogenic vancomycin-resistant *Enterococcus* leading the microbiota reconstitution after FMT in humans. Interestingly, many of the genera found in the PF core at 15 dpft included genera that have different species commonly associated to swine diseases, such as *Campylobacter*^53^*, Clostridium*^54^ and *Actinobacillus*^55^*.* Together with the already-mentioned exclusively members, each one of the core genera found in at least in AWF, deserve further investigation to elucidate the potential role in ASF resistance, since the complexity of the whole microbial network and their interactions^56^ may be essential to promote this effect.

Transplantation of microbiota components from one species to another is also a human ancient practice, since our species has been feeding with a complex community of bacteria composing the milk from diverse animals^57^. A more sophisticated and modern microbiota transplantation in humans is the intake of probiotics such as *Bifidobacterium* or *Lactobacillus* isolated from different animal species^58^. Considering this information, we propose identifying individual components of the warthog microbiota to characterize their protective potential against ASFV. If confirmed the benefits of their administration, we should be able to unravel the mechanisms of action and their potential future use in pigs as probiotics.

As described for humans, FMT improves intestine metabolism, epithelial barrier integrity^33^, performance on suckling piglet^37^, and mucosal immunity even in distal places^59,60^. In brief, the alpha diversity showed a significant increase at 15 dpft for AWF, suggesting a better gut health status in this group^15,24^. The increase in total IgA found in pigs transplanted with warthog feces support this observation. The fact that IgA and IgA + plasma cells play key roles not only in mucosal immunity and gut microbiota composition^61^, but also regulating the innate and adaptive T-cell immunity^62^, could explain the protection afforded against E75CV1 infection. As an example, IgA + cells are capable of inducing the expression of IFN-γ in a TNF-α dependent manner, both cytokines known to play important roles in protection against ASFV^63^. More recent results obtained in the laboratory confirms that transplantation of warthog fecal microbiota components specifically stimulates mucosal immunity in the respiratory track (not shown). With this new evidence at hand, in the near future we plan to change our intramuscular ASFV-challenge model to an in-contact infection protocol, therefore increasing the options to control ASFV infection at the entry site, using either attenuated or virulent ASFV strains.

## Methods

### Animals and animal housing

Forty-eight piglets (Landrace × Large White) were acquired in a commercial farm that was negative for PRRSV, Aujezsky's disease virus, *Pasteurella multocida* and *Brachyspira hyodysenteriae.* At weaning, 3-week-old animals were vaccinated against PCV2 and *Mycoplasma hyopneumoniae* (Porcilis PCV M hyo, MSD Animal Health) and transferred to the animal facilities from the Servei de Granges i Camps Experimentals of the UAB. In these facilities, animals were separated in 4 groups of 6 piglets, placed in 4 independent boxes with individual ventilation and maintained in a 23-25ºC atmosphere. The experiment had a duration of 30 days and all personnel changed clothes, boots and gloves before entering in each box and handling the animals. Piglets were daily inspected for clinical signs. Water and feed were supplied ad libitum. A commercial feed for weaned piglets was provided (P-120, LA GIRONINA, Spain), with essential requirements of ZnO (110 mg/Kg) and without antibiotics.

### Fecal microbiota transplantation

To prepare the FMT, fresh feces were collected from a colony of eleven 4-to-8 years-old warthogs (*Phacochoerus africanus*), from the Barcelona zoo. The colony was originated mating 2 sows with 2 different boars. Warthogs from the zoo were fed with commercial cereal-based feed complemented with apples, potatoes and carrots. No antibiotic treatment was used at least three months prior to the collection of feces. Five different feces droppings were collected in sterile containers from the pen ground within the hour after defecation and stored at 4 °C. Next, 12 g of feces per animal were immersed in 40 ml of buffer protective solution (PBS 2x, glycerol 15% and cysteine 0.1%), and stored at − 80 °C.

Fresh feces were also collected from 5 healthy domestic adult sows from a PRRS-negative PCV2-vaccinated commercial farm. Fecal collection, dilution and storage was done as previously described. Warthog and domestic pig feces were confirmed negative for PRRSV by using a commercial qRT-PCR system (LSI VetMAX PRRSV EU/NA 96 Real-Time PCR Kit, THERMOFISHER).

All the animals were orally inoculated during 3 consecutive days with freshly prepared fecal material processed as follows. Pools of feces from 5 warthogs and from 5 domestic pigs (2 g/animal) were mixed in 40 ml sterile PBS using a commercial vortex machine (IUL) and maintained in special bag containers (stomacher lab system) until homogenized. The fecal slurry was filtered in sterilized gauze to remove larger particles and the filtered feces suspension was dispensed in 50 ml aliquots and kept at 4 °C until inoculation. Ten ml of PBS or freshly prepared feces resuspension were administered into the esophagus through the mouth, by using a 10 cm-long plastic cannula. One group was inoculated with PBS as a control (PBS), the second group was inoculated with domestic pig feces supernatant (pPF) and the third group was inoculated with warthog feces supernatant (pWF). One day before inoculation with warthog feces supernatant, a fourth group (AWF, #37–48) was orally given a dose of a cocktail of antibiotics composed of colistine (CEVA Sante animale; 700,000 UI/kg), neomicine (S.P. Veterinaria; 420 UI/Kg), bacitracine (Alpharma ZOETIS; 420 UI/kg), oxitetraciclina (MAYMO; 0.28 g/kg), and the following day they were inoculated with warthog feces supernatant during 3 consecutive days.

### Fecal sample collection

Fecal samples were collected at 0, 8, 15 days post fecal microbiota transplantation (dpft) and kept at − 80 °C for further processing. Pigs were weighted at 0, 15 and 30 dpft. Rectal temperature was recorded at 0, 4, 8, 15, 22 and 30 dpft. Blood samples were collected in EDTA tubes at 4 and 15 dpft for whole blood cell counting from all pigs. At 30 dpft, 6 animals per group were selected and moved to the BSL-3 facility to perform the ASFV challenge.

### 16S rRNA analysis from fecal samples

DNA was extracted from feces (300 mg per sample) collected from pools of warthog and domestic pig feces used as inoculum (pWF and pPF, respectively), and from feces collected from the WF group (#25, #28, #29, #30, #36) and PBS group ( #1, #3, #4, #9, #11) at 15 dpft. Moreover, DNA was extracted from feces collected on 0, 8 and 15 dpft for groups PF (#13, #16, #18, #22, #24) and AWF (#37, #41, #43, #44, #46). Briefly, frozen feces were suspended in 900 µl PBS by vortex. After centrifuging at 12,000 rpm/min for 10 min, 200 µl of the supernatant were submitted to genomic DNA extraction using Machinery Nigel Kit (GmbH & Co, Düren; Germany). Purified DNA was eluted in a final volume of 50 µl elution buffer. The quality and quantity of genomic DNA was evaluated on a BioDrop DUO (BioDrop Ltd).

The V3-V4 region of the 16S rRNA gene (~ 460 bp) was targeted^64^ to perform amplification and sequencing using Illumina pair-end 2 × 250 bp sequencing with MiSeq, following the manufacturer instructions (MS-102–2003 Miseq Reagent Kit v2,500 cycle). Sequence reads were submitted to quality control using FastQC software^65^. The QIIME^66,67^ software package (version 2019.10) was used to process the reads and infer the microbiota composition. Denoising and trimming was done with DADA2^68^ under the default parameters to exclude both primers and low-quality reads from the sequences. Taxonomic classification was done with the machine learning Pyhton library scikit^67–69^ using the pre-trained naïve Bayes classifier trained against Greengenes^70^ (gg-13-8-99-nt-classifier) provided by qiime2 project (available at https://docs.qiime2.org/). The core-taxa was calculated with *in-house* scripts to find the taxa present in all animals from a group at a particular timepoint. Phylogeny was built aligning reads using MAFFT^71^ masking reads to remove not-conserved positions and building a tree rooted with FastTree2^72^. Rarefaction was done to evaluate the depth of sampling. Alpha and beta-diversity metrics were calculated at maximum depth. Shannon^73^ and Chao indexes^74^ were estimated as measurements of the alpha diversity and richness of the samples, respectively. Alpha diversity between groups was compared through two-sample non-parametric t-tests (Monte Carlo method) at maximum depth in rarefied samples (with 999 permutations). Unifrac weighted and unweighted distances were calculated to assess differences across samples^75,76^. Principal coordinates analysis (PCoA) was done to visualize the distances or dissimilarities matrices. Venn graphs were done using Venn diagram software (available at https://bioinformatics.psb.ugent.be/webtools/Venn/).

### Hematology

Blood collected in EDTA tubes was analyzed for whole blood count in the Servei d’Hematologia Clínica Veterinària of the UAB (ADVIA 120, Siemens). Laboratory Reference Values were according to previous reports^77^.

### Histopathology

A total of fifteen pigs: four from the PBS group (#6, #8, #10, #12), five from the WF group (#26, #27, #31, #34, #35), three from the PF group (#14, #19, #20), and three from the AWF group (#39, #45, #47), were sacrificed 30 dpft for a comparative morphometric study using both small intestine and colon samples. The procedure followed is based on a methodology previously described^78^ with slight modifications. Briefly, tissues were dehydrated and embedded in paraffin wax, sectioned at 3 µm, and stained with hematoxylin and eosin (HE). Villi height and crypt depth from ileum and crypt depth from colon were assessed on 10 well-oriented villi and crypts for each animal. Villus:crypt ratio was assessed by dividing villus height by crypt depth. Sections were analyzed under the light microscope in a blind-fashion manner by one only person.

### Quantification of total IgA

The concentration of secretory IgA was measured in pig sera using the porcine ELISA kit following the manufacter’s recommendations (E101-102; Bethyl Laboratories, Inc., Montgomery).

### African swine fever virus experimental infection after FMT

At the end of the FMT experiment, 6 pigs from each group were selected for the ASF experimental infection and moved into a BSL-3 facility. After a three-day period of adaptation, pigs from PBS and WF were intramuscularly inoculated with the E75 virulent strain (10^4^ HAU); while pigs from PF and AWF were intramuscularly injected with 100 HAU of the attenuated E75CV1 strain, aiming to facilitate the observation of any potential antiviral effect^20^. Water and feed were supplied ad libitum with a commercial feed for growing piglets (Feed N. 555 growing pigs, Corporación alimentaria Guissona S.A., Lleida, Spain), which contained ZnO (94 ppm/kg) as an addition.

Temperature and clinical signs were daily recorded. Clinical scores were calculated following a guide previously published by the group^27^. Serum and nasal swabs were taken at 0, 4, 7, 13 and 24 days post-infection (dpi). Serum were used for checking specific antibody responses against ASFV by ELISA^20^, serum and nasal swabs were checked by qPCR for the virus excretion^20^. PBMCs were extracted on 13 and 24 dpi for monitoring specific T‑cell responses by ELISPOT^20^.

### Statistical analysis

Statistical analysis was performed using SPSS 17.0 software (SPSS Inc, Chicago, USA). Differences in hematology, animal weight and daily weight gain during FMT were analyzed by one-way ANOVA.

The significant differences in alpha diversity were evaluated through Kruskal–Wallis test^79^. The percentage of variation between grouped samples was measured by R2, using Adonis function of the vegan package in R software^80^. Estimation of P values was done through Monte Carlo test with 999 random permutations of the data set. Permutational analysis of the variance (PERMANOVA) was performed to compare beta diversity matrices over the treatments under study with 999 permutations (*q2 diversity beta-group-significance*)^81^. To identify differentially abundant taxa from the microbiota from different groups and timepoints, analysis of composition of microbiomes (ANCOM)^82^ was done using the *qiime composition ancom* plugin from QIIME.

When *P values* were *P* < 0.05 (*) or *P* < 0.01 (**) they were considered significantly different, *P* < 0.005 (***) was considered highly different, while *P* < 0.10 referred to a trend of showing statistically difference.

### Ethics statement

All experiments were performed in the Servei de Granges i Camps Experimentals of the Universitat Autònoma de Barcelona (UAB) and the Biosafety Level 3 facilities of the Centre de Recerca en Sanitat Animal (IRTA-CReSA, Barcelona). Animal care and experiments were performed in accordance to relevant guidelines and regulations, including the Good Experimental Practices (GEP) guidelines. All procedures were done under the approval of the Ethical and Animal Welfare Committee of the UAB (Permit Number: CEEAH 3166).

## Supplementary information

Supplementary Information.

## Data Availability

The entire sequence dataset is available in the NCBI database, BioProject PRJNA625746 and BioSamples SAMN14608419-14608463.
