# Supplementary material for: Fecal microbiota transplantation from warthog to pig confirms the influence of the gut microbiota on African swine fever susceptibility
Source: Sci Rep. 2020 Oct 19;10:17605. doi: 10.1038/s41598-020-74651-3 (PMC7573625; doi:10.1038/s41598-020-74651-3)
Supplement: Supplementary file 1 — Supplementary Information. [file 41598_2020_74651_MOESM1_ESM.pdf]

Fecal microbiota transplantation from warthog to pig confirms the influence of the gut microbiota on African swine fever susceptibility

Jinya Zhang[1], Fernando Rodríguez[1,*], Maria Jesus Navas[1], Mar Costa-Hurtado[1], Vanessa[3] Almagro, Laia Bosch-Camós[1], Elisabeth López[1], Raul Cuadrado[1], Francesc Accensi[1,2], Sonia Pina-Pedrero[1], Jorge Martínez[1,2,@], Florencia Correa-Fiz[1,@,*]

[1]IRTA, Centre de Recerca en Sanitat Animal (CReSA, IRTA), Campus de la Universitat Autònoma de Barcelona, 08193 Bellaterra, Spain.

[2]Departament de Sanitat i Anatomia Animals, Universitat Autònoma de Barcelona. 08193, Bellaterra, Barcelona, Spain

[3]Veterinary service Zoo Barcelona, Parc Ciudadella s/n 08003, Barcelona, Spain.

Supplementary Table S1. Number of sequences per sample obtained after filtering used for this study.

Sample ID (#animal_dpft)	Sequence Count
13D15	1,425
1D15	39,774
3D15	34,841
4D15	11,643
6D15	48,778
9D15	62,010
16D15	44,153
18D15	51,097
22D15	55,039
24D15	23,711
25D15	26,415
28D15	30,556
29D15	43,867
30D15	36,541
36D15	27,450
37D15	23,849
41D15	53,813
43D15	67,027
44D15	29,913
46D15	19,050

Sample ID (pools)	Sequence Count
pWF	81,614
pPF	53,689

Sample ID (#animal_dpft)	Sequence Count
13D0	47,310
16D0	50,364
18D0	24,635
22D0	39,719
24D0	45,343
37D0	38,801
41D0	46,035
43D0	43,323
44D0	30,032
46D0	38,076

Supplementary Table S1. Number of sequences per sample obtained after filtering used for this study (cont.).

Sample ID (#animal_dpft)	Sequence Count
13D8	25,989
16D8	44,081
18D8	32,428
22D8	40,595
24D8	15,640
37D8	19,232
41D8	38168
43D8	16,850
44D8	10,054
46D8	26,878

Supplementary Table S2. Relative abundance (%) of taxa assigned at different levels (Phyla, Family and Genera) for each animal sample and mean relative abundance (%) for each group of animals.

		PF_15dpft					WF_15dpft						AWF_15dpft						PBS_15dpft					
Kingdom	Phyla	16	18	22	24	AVERAGE	25	28	29	30	36	AVERAGE	37	41	43	44	46	AVERAGE	1	3	4	9	11	AVERAGE
Bacteria	*Actinobacteria*	0,000	0,149	0,220	0,169	0,134	0,000	0,000	0,217	0,000	0,204	0,084	0,117	0,000	0,179	0,057	0,037	0,078	0,000	0,000	0,000	0,000	0,103	0,021
Bacteria	*Bacteroidetes*	18,590	17,825	24,712	29,147	22,568	26,163	15,038	30,996	17,952	21,322	22,294	26,399	15,649	20,486	41,484	29,291	26,662	20,395	16,128	12,282	19,267	14,780	16,570
Bacteria	*Chlamydiae*	0,000	0,168	0,000	0,000	0,042	0,000	0,383	0,000	0,000	0,521	0,181	0,583	0,106	0,145	0,204	1,475	0,502	0,221	0,000	0,000	0,000	0,116	0,067
Bacteria	*Chloroflexi*	0,000	0,000	0,000	0,000	0,000	0,000	0,000	0,000	0,000	0,000	0,000	0,000	0,000	0,000	0,000	0,016	0,003	0,000	0,000	0,000	0,000	0,000	0,000
Bacteria	*Cyanobacteria*	0,283	0,618	0,325	0,198	0,356	0,000	0,121	0,552	0,501	0,197	0,274	0,021	0,136	0,275	0,000	0,100	0,106	0,128	0,218	0,000	0,935	0,137	0,284
Bacteria	*Deferribacteres*	0,116	0,061	0,000	0,017	0,048	0,129	0,000	0,000	0,000	0,342	0,094	0,109	0,098	0,136	0,097	0,000	0,088	0,000	0,000	0,000	0,357	0,060	0,083
Bacteria	*Elusimicrobia*	0,000	0,029	0,031	0,000	0,015	0,011	0,000	0,000	0,071	0,120	0,041	0,000	0,000	0,000	0,007	0,315	0,064	0,000	0,000	0,000	0,000	0,035	0,007
Archaea	*Euryarchaeota*	0,000	0,000	0,124	0,257	0,095	0,000	0,000	0,000	0,000	0,058	0,012	0,294	0,000	0,069	0,057	0,278	0,139	0,000	0,000	0,000	0,000	0,024	0,005
Bacteria	*Fibrobacteres*	0,000	0,029	0,000	0,101	0,033	0,000	0,000	0,098	0,000	0,000	0,020	0,000	0,279	0,064	0,000	0,152	0,099	0,000	0,000	0,000	0,000	0,189	0,038
Bacteria	*Firmicutes*	69,123	70,624	55,619	50,673	61,510	61,064	83,178	60,932	49,646	63,333	63,631	52,891	70,446	66,267	48,130	54,908	58,528	65,309	73,029	76,226	52,015	57,191	64,754
Bacteria	*Fusobacteria*	0,000	0,000	0,000	0,000	0,000	0,000	0,000	0,000	0,000	0,000	0,000	0,000	0,000	0,000	0,000	0,000	0,000	0,000	0,000	0,000	0,000	0,000	0,000
Bacteria	*Lentisphaerae*	0,358	0,131	0,104	0,000	0,148	1,049	0,000	0,506	0,213	0,244	0,402	0,084	0,210	0,149	0,000	0,110	0,111	0,000	0,583	0,000	0,154	0,000	0,147
Bacteria	*OD1*	0,000	0,000	0,000	0,000	0,000	0,000	0,000	0,000	0,000	0,000	0,000	0,000	0,000	0,000	0,000	0,000	0,000	0,000	0,000	0,000	0,000	0,000	0,000
Bacteria	*Planctomycetes*	0,000	0,000	0,000	0,000	0,000	0,000	0,000	0,000	0,000	0,000	0,000	0,000	0,000	0,000	0,000	0,000	0,000	0,000	0,000	0,000	0,000	0,000	0,000
Bacteria	*Proteobacteria*	1,649	0,742	2,353	1,101	1,461	0,216	0,507	1,040	2,230	2,200	1,239	3,489	0,433	1,599	1,464	0,766	1,550	0,832	0,944	0,000	4,506	0,848	1,426
Bacteria	*Spirochaetes*	1,619	0,613	1,446	2,029	1,427	0,541	0,000	0,513	0,364	0,066	0,297	2,252	1,646	1,513	1,936	2,835	2,036	0,028	0,057	0,000	0,119	0,703	0,181
Bacteria	*Synergistetes*	0,000	0,000	0,000	0,000	0,000	0,000	0,000	0,000	0,000	0,000	0,000	0,000	0,000	0,000	0,000	0,000	0,000	0,000	0,000	0,000	0,000	0,000	0,000
Bacteria	*Tenericutes*	0,000	0,086	0,233	1,485	0,451	0,053	0,000	0,000	0,000	0,109	0,032	1,413	0,299	0,179	0,114	0,115	0,424	0,040	0,043	0,000	0,086	0,010	0,036
Bacteria	*TM7*	0,231	0,646	0,603	0,789	0,567	0,000	0,000	0,000	0,000	0,761	0,152	0,138	0,983	0,315	0,528	0,667	0,526	0,644	0,373	0,000	0,310	0,221	0,309
Bacteria	*Unclassified*	7,698	7,910	13,470	13,466	10,636	10,774	0,772	4,938	28,965	10,226	11,135	11,497	8,375	8,153	5,770	8,934	8,546	12,222	7,818	11,492	22,112	25,472	15,823
Bacteria	*Unclassified*	0,152	0,110	0,420	0,333	0,254	0,000	0,000	0,210	0,057	0,295	0,112	0,021	0,544	0,136	0,154	0,000	0,171	0,181	0,772	0,000	0,139	0,077	0,234
Bacteria	*Verrucomicrobia*	0,181	0,258	0,342	0,236	0,254	0,000	0,000	0,000	0,000	0,000	0,000	0,692	0,795	0,336	0,000	0,000	0,365	0,000	0,034	0,000	0,000	0,034	0,014

Supplementary Table S2. Relative abundance (%) of taxa assigned at different levels (Phyla, Family and Genera) for each animal sample and mean relative abundance (%) for each group of animals(cont.).

Kingdom	Phyla	Class	Order	Family	PF_15dpft					WF_15dpft						AWF_15dpft						PBS_15dpft					
					16	18	22	24	AVERAGE	25	28	29	30	36	AVERAGE	37	41	43	44	46	AVERAGE	1	3	4	9	11	AVERAGE
Bacteria	*Bacteroidetes*	*Bacteroidia*	*Bacteroidales*	*[Barnesiellaceae]*	0,303	0,546	0,000	1,088	0,484	0,572	0,507	0,030	0,000	0,368	0,295	0,805	0,894	0,655	2,898	0,835	1,217	0,000	0,000	0,000	0,000	0,000	0,000
Bacteria	*Verrucomicrobia*	*Opitutae*	*[Cerasicoccales]*	*[Cerasicoccaceae]*	0,000	0,000	0,000	0,000	0,000	0,000	0,000	0,000	0,000	0,000	0,000	0,000	0,000	0,000	0,000	0,000	0,000	0,000	0,000	0,000	0,000	0,000	0,000
Bacteria	*Verrucomicrobia*	*[Spartobacteria]*	*[Chthoniobacterales]*	*[Chthoniobacteraceae]*	0,000	0,000	0,000	0,000	0,000	0,000	0,000	0,000	0,000	0,000	0,000	0,000	0,000	0,000	0,000	0,000	0,000	0,000	0,034	0,000	0,000	0,000	0,007
Archaea	*Euryarchaeota*	*Thermoplasmata*	*E2*	*[Methanomassiliicoccaceae]*	0,000	0,000	0,124	0,224	0,087	0,000	0,000	0,000	0,000	0,058	0,012	0,180	0,000	0,069	0,057	0,110	0,083	0,000	0,000	0,000	0,000	0,024	0,005
Bacteria	*Bacteroidetes*	*Bacteroidia*	*Bacteroidales*	*[Odoribacteraceae]*	0,000	0,000	0,000	0,000	0,000	0,000	0,000	0,000	0,000	0,000	0,000	0,000	0,000	0,000	0,000	0,000	0,000	0,000	0,000	0,000	0,000	0,000	0,000
Bacteria	*Bacteroidetes*	*Bacteroidia*	*Bacteroidales*	*[Paraprevotellaceae]*	2,016	0,810	1,513	1,518	1,464	0,644	0,874	0,857	1,355	0,528	0,851	1,610	0,927	0,830	2,501	2,199	1,613	1,222	0,376	0,000	3,377	1,082	1,211
Bacteria	*Firmicutes*	*Clostridia*	*Clostridiales*	*[Tissierellaceae]*	0,000	0,000	0,000	0,000	0,000	0,000	0,000	0,000	0,000	0,000	0,000	0,000	0,000	0,000	0,000	0,000	0,000	0,000	0,000	0,000	0,000	0,000	0,000
Bacteria	*Bacteroidetes*	*Flavobacteriia*	*Flavobacteriales*	*[Weeksellaceae]*	0,000	0,000	0,000	0,000	0,000	0,000	0,000	0,000	0,000	0,000	0,000	0,000	0,000	0,000	0,000	0,000	0,000	0,068	0,000	0,000	0,047	0,000	0,023
Bacteria	*Proteobacteria*	*Alphaproteobacteria*	*Rhodospirillales*	*Acetobacteraceae*	0,000	0,000	0,000	0,000	0,000	0,000	0,000	0,000	0,019	0,000	0,004	0,000	0,000	0,000	0,000	0,000	0,000	0,000	0,000	0,000	0,000	0,000	0,000
Bacteria	*Tenericutes*	*Mollicutes*	*Acholeplasmatales*	*Acholeplasmataceae*	0,000	0,086	0,042	0,540	0,167	0,053	0,000	0,000	0,000	0,000	0,011	0,273	0,171	0,000	0,114	0,115	0,135	0,000	0,000	0,000	0,086	0,010	0,019
Bacteria	*Proteobacteria*	*Betaproteobacteria*	*Burkholderiales*	*Alcaligenaceae*	0,000	0,000	0,000	0,000	0,000	0,000	0,000	0,000	0,000	0,000	0,000	0,000	0,000	0,000	0,000	0,000	0,000	0,000	0,000	0,000	0,000	0,000	0,000
Bacteria	*Tenericutes*	*Mollicutes*	*Anaeroplasmatales*	*Anaeroplasmataceae*	0,000	0,000	0,000	0,135	0,034	0,000	0,000	0,000	0,000	0,015	0,003	0,008	0,128	0,031	0,000	0,000	0,034	0,000	0,000	0,000	0,000	0,000	0,000
Bacteria	*Firmicutes*	*Bacilli*	*Bacillales*	*Bacillaceae*	0,485	1,180	0,044	0,304	0,503	0,000	0,000	0,000	0,000	0,441	0,088	1,061	0,368	0,291	0,415	0,656	0,558	0,370	0,445	0,000	0,107	0,224	0,229
Bacteria	*Bacteroidetes*	*Bacteroidia*	*Bacteroidales*	*Bacteroidaceae*	0,000	0,000	0,000	0,000	0,000	0,000	0,000	0,089	0,052	0,128	0,054	0,000	0,020	0,000	0,000	0,000	0,004	0,096	0,112	0,000	0,103	0,024	0,067
Bacteria	*Actinobacteria*	*Actinobacteria*	*Bifidobacteriales*	*Bifidobacteriaceae*	0,000	0,000	0,000	0,000	0,000	0,000	0,000	0,000	0,000	0,000	0,000	0,000	0,000	0,000	0,000	0,000	0,000	0,000	0,000	0,000	0,000	0,000	0,000
Bacteria	*Spirochaetes*	*[Brachyspirae]*	*[Brachyspirales]*	*Brachyspiraceae*	0,000	0,000	0,000	0,000	0,000	0,000	0,000	0,000	0,000	0,000	0,000	0,000	0,000	0,000	0,000	0,000	0,000	0,000	0,000	0,000	0,000	0,000	0,000
Bacteria	*Proteobacteria*	*Epsilonproteobacteria*	*Campylobacterales*	*Campylobacteraceae*	0,131	0,047	0,067	0,245	0,123	0,000	0,000	0,367	0,131	0,692	0,238	0,084	0,000	0,224	0,000	0,000	0,062	0,123	0,077	0,000	0,568	0,032	0,160
Bacteria	*Proteobacteria*	*Alphaproteobacteria*	*Caulobacterales*	*Caulobacteraceae*	0,000	0,000	0,000	0,000	0,000	0,000	0,000	0,000	0,000	0,000	0,000	0,000	0,000	0,000	0,017	0,000	0,003	0,000	0,000	0,000	0,000	0,000	0,000
Bacteria	*Bacteroidetes*	*[Saprospirae]*	*[Saprospirales]*	*Chitinophagaceae*	0,000	0,000	0,024	0,000	0,006	0,000	0,000	0,000	0,000	0,000	0,000	0,000	0,000	0,012	0,000	0,000	0,002	0,000	0,000	0,000	0,000	0,000	0,000
Bacteria	*Chlamydiae*	*Chlamydiia*	*Chlamydiales*	*Chlamydiaceae*	0,000	0,168	0,000	0,000	0,042	0,000	0,383	0,000	0,000	0,521	0,181	0,583	0,106	0,145	0,204	1,475	0,502	0,221	0,000	0,000	0,000	0,102	0,065
Bacteria	*Firmicutes*	*Clostridia*	*Clostridiales*	*Christensenellaceae*	0,000	0,000	0,000	0,000	0,000	0,000	0,000	0,000	0,000	0,000	0,000	0,000	0,000	0,000	0,000	0,084	0,017	0,000	0,000	0,000	0,000	0,000	0,000
Bacteria	*Firmicutes*	*Clostridia*	*Clostridiales*	*Clostridiaceae*	1,964	11,281	0,899	1,396	3,885	3,759	0,471	2,113	0,290	2,645	1,856	3,686	3,455	2,245	3,818	1,512	2,943	5,579	2,003	8,701	0,000	1,069	3,470
Bacteria	*Proteobacteria*	*Betaproteobacteria*	*Burkholderiales*	*Comamonadaceae*	0,000	0,029	0,000	0,000	0,007	0,000	0,141	0,041	0,000	0,084	0,053	0,000	0,015	0,018	0,053	0,000	0,017	0,070	0,000	0,000	0,000	0,000	0,014
Bacteria	*Actinobacteria*	*Coriobacteriia*	*Coriobacteriales*	*Coriobacteriaceae*	0,000	0,000	0,000	0,000	0,000	0,000	0,000	0,000	0,000	0,000	0,000	0,000	0,000	0,000	0,000	0,000	0,000	0,000	0,000	0,000	0,000	0,000	0,000
Bacteria	*Deferribacteres*	*Deferribacteres*	*Deferribacterales*	*Deferribacteraceae*	0,116	0,061	0,000	0,017	0,048	0,129	0,000	0,000	0,000	0,342	0,094	0,109	0,098	0,136	0,097	0,000	0,088	0,000	0,000	0,000	0,357	0,060	0,083
Bacteria	*Proteobacteria*	*Deltaproteobacteria*	*Desulfovibrionales*	*Desulfovibrionaceae*	0,267	0,084	0,116	0,000	0,117	0,000	0,157	0,036	0,047	0,313	0,111	0,046	0,061	0,048	0,084	0,084	0,065	0,103	0,000	0,000	0,000	0,045	0,030
Bacteria	*Synergistetes*	*Synergistia*	*Synergistales*	*Dethiosulfovibrionaceae*	0,000	0,000	0,000	0,000	0,000	0,000	0,000	0,000	0,000	0,000	0,000	0,000	0,000	0,000	0,000	0,000	0,000	0,000	0,000	0,000	0,000	0,000	0,000
Bacteria	*Actinobacteria*	*Actinobacteria*	*Actinomycetales*	*Dietziaceae*	0,000	0,000	0,000	0,000	0,000	0,000	0,000	0,000	0,000	0,000	0,000	0,059	0,000	0,000	0,000	0,000	0,012	0,000	0,000	0,000	0,000	0,000	0,000
Bacteria	*Elusimicrobia*	*Elusimicrobia*	*Elusimicrobiales*	*Elusimicrobiaceae*	0,000	0,029	0,031	0,000	0,015	0,011	0,000	0,000	0,071	0,120	0,041	0,000	0,000	0,000	0,007	0,315	0,064	0,000	0,000	0,000	0,000	0,035	0,007
Bacteria	*Proteobacteria*	*Gammaproteobacteria*	*Enterobacteriales*	*Enterobacteriaceae*	0,000	0,000	0,000	0,000	0,000	0,000	0,000	0,057	0,000	0,321	0,076	0,423	0,173	0,230	0,284	0,000	0,222	0,000	0,000	0,000	0,000	0,000	0,000
Bacteria	*Tenericutes*	*Mollicutes*	*Entomoplasmatales*	*Entomoplasmataceae*	0,000	0,000	0,000	0,000	0,000	0,000	0,000	0,000	0,000	0,000	0,000	0,000	0,000	0,000	0,000	0,000	0,000	0,000	0,000	0,000	0,000	0,000	0,000
Bacteria	*Firmicutes*	*Erysipelotrichi*	*Erysipelotrichales*	*Erysipelotrichaceae*	8,378	7,771	4,297	4,960	6,351	6,292	15,051	7,215	7,414	10,444	9,283	5,472	8,362	13,074	3,901	6,320	7,426	8,649	17,273	28,343	3,776	11,906	13,989
Bacteria	*Firmicutes*	*Clostridia*	*Clostridiales*	*Eubacteriaceae*	0,000	0,000	0,000	0,000	0,000	0,000	0,000	0,000	0,000	0,000	0,000	0,000	0,000	0,037	0,000	0,063	0,020	0,000	0,000	0,000	0,000	0,060	0,012
Bacteria	*TM7*	*TM7-3*	*CW040*	*F16*	0,231	0,646	0,603	0,789	0,567	0,000	0,000	0,000	0,000	0,761	0,152	0,138	0,983	0,315	0,528	0,667	0,526	0,644	0,373	0,000	0,310	0,221	0,309
Bacteria	*Chloroflexi*	*TK10*	*B07_WMSP1*	*FFCH4570*	0,000	0,000	0,000	0,000	0,000	0,000	0,000	0,000	0,000	0,000	0,000	0,000	0,000	0,000	0,000	0,016	0,003	0,000	0,000	0,000	0,000	0,000	0,000
Bacteria	*Fibrobacteres*	*Fibrobacteria*	*Fibrobacterales*	*Fibrobacteraceae*	0,000	0,029	0,000	0,101	0,033	0,000	0,000	0,098	0,000	0,000	0,020	0,000	0,279	0,064	0,000	0,152	0,099	0,000	0,000	0,000	0,000	0,189	0,038
Bacteria	*Bacteroidetes*	*Flavobacteriia*	*Flavobacteriales*	*Flavobacteriaceae*	0,000	0,000	0,000	0,013	0,003	0,000	0,000	0,000	0,000	0,000	0,000	0,008	0,017	0,000	0,000	0,000	0,005	0,000	0,000	0,000	0,000	0,000	0,000
Bacteria	*Fusobacteria*	*Fusobacteriia*	*Fusobacteriales*	*Fusobacteriaceae*	0,000	0,000	0,000	0,000	0,000	0,000	0,000	0,000	0,000	0,000	0,000	0,000	0,000	0,000	0,000	0,000	0,000	0,000	0,000	0,000	0,000	0,000	0,000
Bacteria	*Actinobacteria*	*Actinobacteria*	*Actinomycetales*	*Gordoniaceae*	0,000	0,000	0,000	0,000	0,000	0,000	0,000	0,000	0,000	0,000	0,000	0,000	0,000	0,000	0,000	0,000	0,000	0,000	0,000	0,000	0,000	0,000	0,000
Bacteria	*Proteobacteria*	*Epsilonproteobacteria*	*Campylobacterales*	*Helicobacteraceae*	0,000	0,000	0,000	0,139	0,035	0,106	0,000	0,000	0,183	0,000	0,058	0,159	0,000	0,210	0,274	0,000	0,129	0,111	0,049	0,000	0,324	0,000	0,097
Bacteria	*Firmicutes*	*Clostridia*	*Clostridiales*	*Lachnospiraceae*	1,862	2,626	1,848	2,480	2,204	1,885	0,818	2,685	1,407	5,993	2,558	1,308	3,945	2,960	2,822	3,218	2,851	1,773	2,804	1,615	2,809	2,153	2,231
Bacteria	*Firmicutes*	*Bacilli*	*Lactobacillales*	*Lactobacillaceae*	0,000	0,020	0,489	0,000	0,127	0,000	0,052	0,255	0,060	0,000	0,074	0,092	0,000	0,010	0,000	0,000	0,021	0,148	0,000	0,000	0,000	0,226	0,075
Bacteria	*Bacteroidetes*	*Bacteroidia*	*Bacteroidales*	*Marinilabiaceae*	0,000	0,000	0,000	0,000	0,000	0,000	0,000	0,000	0,000	0,000	0,000	0,000	0,000	0,000	0,000	0,000	0,000	0,000	0,000	0,000	0,000	0,000	0,000
Archaea	*Euryarchaeota*	*Methanobacteria*	*Methanobacteriales*	*Methanobacteriaceae*	0,000	0,000	0,000	0,034	0,008	0,000	0,000	0,000	0,000	0,000	0,000	0,113	0,000	0,000	0,000	0,168	0,056	0,000	0,000	0,000	0,000	0,000	0,000
Bacteria	*Actinobacteria*	*Actinobacteria*	*Actinomycetales*	*Microbacteriaceae*	0,000	0,000	0,000	0,000	0,000	0,000	0,000	0,000	0,000	0,000	0,000	0,000	0,000	0,000	0,037	0,000	0,007	0,000	0,000	0,000	0,000	0,000	0,000
Bacteria	*Actinobacteria*	*Actinobacteria*	*Actinomycetales*	*Micrococcaceae*	0,000	0,037	0,000	0,000	0,009	0,000	0,000	0,000	0,000	0,000	0,000	0,000	0,000	0,000	0,000	0,000	0,000	0,000	0,000	0,000	0,000	0,000	0,000
Bacteria	*Proteobacteria*	*Gammaproteobacteria*	*Pseudomonadales*	*Moraxellaceae*	0,000	0,084	0,000	0,000	0,021	0,110	0,000	0,091	0,036	0,186	0,084	0,000	0,009	0,200	0,017	0,000	0,045	0,146	0,255	0,000	0,051	0,000	0,091
Bacteria	*Tenericutes*	*Mollicutes*	*Mycoplasmatales*	*Mycoplasmataceae*	0,000	0,000	0,000	0,000	0,000	0,000	0,000	0,000	0,000	0,000	0,000	0,059	0,000	0,148	0,000	0,000	0,041	0,040	0,043	0,000	0,000	0,000	0,017
Bacteria	*Proteobacteria*	*Betaproteobacteria*	*Neisseriales*	*Neisseriaceae*	0,000	0,000	0,000	0,000	0,000	0,000	0,000	0,000	0,000	0,000	0,000	0,000	0,000	0,000	0,020	0,000	0,004	0,000	0,000	0,000	0,000	0,000	0,000
Bacteria	*Proteobacteria*	*Betaproteobacteria*	*Burkholderiales*	*Oxalobacteraceae*	0,238	0,000	0,187	0,000	0,106	0,000	0,000	0,000	0,369	0,128	0,099	0,491	0,000	0,294	0,067	0,215	0,213	0,035	0,000	0,000	0,207	0,000	0,048
Bacteria	*Bacteroidetes*	*Bacteroidia*	*Bacteroidales*	*p-2534-18B5*	0,113	0,272	1,781	0,308	0,618	0,083	0,000	0,486	0,427	0,000	0,199	1,874	0,405	0,116	0,856	0,562	0,763	0,000	0,000	0,000	1,474	0,000	0,295
Bacteria	*Proteobacteria*	*Gammaproteobacteria*	*Pasteurellales*	*Pasteurellaceae*	0,036	0,061	0,027	0,114	0,060	0,000	0,118	0,048	0,000	0,040	0,041	0,075	0,000	0,093	0,000	0,000	0,034	0,000	0,000	0,000	0,000	0,042	0,008
Bacteria	*Firmicutes*	*Clostridia*	*Clostridiales*	*Peptostreptococcaceae*	0,904	3,372	0,601	0,915	1,448	1,564	0,589	1,468	0,085	1,220	0,985	7,057	1,979	2,542	2,955	1,071	3,121	1,916	1,352	2,233	0,402	0,666	1,314
Bacteria	*Cyanobacteria*	*Oscillatoriophycideae*	*Oscillatoriales*	*Phormidiaceae*	0,000	0,000	0,000	0,000	0,000	0,000	0,000	0,000	0,000	0,000	0,000	0,000	0,000	0,000	0,000	0,000	0,000	0,000	0,000	0,000	0,000	0,000	0,000
Bacteria	*Planctomycetes*	*Planctomycetia*	*Pirellulales*	*Pirellulaceae*	0,000	0,000	0,000	0,000	0,000	0,000	0,000	0,000	0,000	0,000	0,000	0,000	0,000	0,000	0,000	0,000	0,000	0,000	0,000	0,000	0,000	0,000	0,000
Bacteria	*Firmicutes*	*Bacilli*	*Bacillales*	*Planococcaceae*	0,000	0,000	0,000	0,000	0,000	0,000	0,000	0,000	0,000	0,000	0,000	0,000	0,000	0,000	0,000	0,042	0,008	0,000	0,000	0,000	0,000	0,000	0,000
Bacteria	*Bacteroidetes*	*Bacteroidia*	*Bacteroidales*	*Porphyromonadaceae*	0,000	0,023	0,042	0,000	0,016	0,000	0,065	0,048	0,022	0,000	0,027	0,000	0,000	0,000	0,047	0,031	0,016	0,000	0,063	0,000	0,082	0,021	0,033
Bacteria	*Bacteroidetes*	*Bacteroidia*	*Bacteroidales*	*Prevotellaceae*	8,070	9,971	7,533	9,316	8,723	13,807	8,797	23,462	8,218	16,098	14,076	11,376	7,359	6,635	14,164	10,047	9,916	16,916	9,483	8,606	5,574	8,820	9,880
Bacteria	*Actinobacteria*	*Actinobacteria*	*Actinomycetales*	*Propionibacteriaceae*	0,000	0,000	0,000	0,000	0,000	0,000	0,000	0,000	0,000	0,000	0,000	0,059	0,000	0,000	0,020	0,037	0,023	0,000	0,000	0,000	0,000	0,000	0,000
Bacteria	*Bacteroidetes*	*Bacteroidia*	*Bacteroidales*	*RF16*	0,000	0,004	0,000	0,000	0,001	0,000	0,000	0,000	0,000	0,000	0,000	0,000	0,000	0,000	0,000	0,000	0,000	0,000	0,000	0,000	0,000	0,000	0,000
Bacteria	*Verrucomicrobia*	*Verruco-5*	*WCHB1-41*	*RFP12*	0,181	0,215	0,307	0,236	0,235	0,000	0,000	0,000	0,000	0,000	0,000	0,692	0,795	0,336	0,000	0,000	0,365	0,000	0,000	0,000	0,000	0,000	0,000
Bacteria	*Proteobacteria*	*Alphaproteobacteria*	*Rhizobiales*	*Rhizobiaceae*	0,000	0,000	0,000	0,000	0,000	0,000	0,000	0,000	0,000	0,000	0,000	0,000	0,000	0,000	0,000	0,000	0,000	0,000	0,000	0,000	0,000	0,000	0,000
Bacteria	*Proteobacteria*	*Betaproteobacteria*	*Rhodocyclales*	*Rhodocyclaceae*	0,000	0,000	0,000	0,000	0,000	0,000	0,000	0,000	0,016	0,051	0,013	0,000	0,000	0,000	0,000	0,000	0,000	0,000	0,000	0,000	0,000	0,000	0,000
Bacteria	*Proteobacteria*	*Alphaproteobacteria*	*Rhodospirillales*	*Rhodospirillaceae*	0,000	0,000	0,581	0,000	0,145	0,000	0,000	0,000	0,000	0,000	0,000	0,168	0,000	0,000	0,000	0,000	0,034	0,000	0,000	0,000	0,849	0,000	0,170
Bacteria	*Bacteroidetes*	*Bacteroidia*	*Bacteroidales*	*Rikenellaceae*	0,041	0,000	0,000	0,000	0,010	0,000	0,056	0,196	0,000	0,087	0,068	0,000	0,000	0,042	0,498	0,079	0,124	0,000	0,029	0,000	0,000	0,027	0,011
Bacteria	*Firmicutes*	*Clostridia*	*Clostridiales*	*Ruminococcaceae*	38,591	25,230	30,362	24,647	29,708	26,511	59,936	27,545	22,750	23,942	32,137	21,301	31,149	29,152	25,190	25,648	26,488	32,111	32,499	29,778	28,865	24,517	29,554
Bacteria	*Bacteroidetes*	*Bacteroidia*	*Bacteroidales*	*S24-7*	0,000	0,000	0,000	0,000	0,000	0,000	0,000	0,000	0,000	0,000	0,000	0,000	0,000	0,000	0,214	0,000	0,043	0,000	0,000	0,000	0,000	0,000	0,000
Bacteria	*Spirochaetes*	*Spirochaetes*	*Sphaerochaetales*	*Sphaerochaetaceae*	0,000	0,000	0,000	0,000	0,000	0,000	0,000	0,000	0,000	0,000	0,000	0,000	0,000	0,000	0,000	0,000	0,000	0,000	0,000	0,000	0,000	0,000	0,000
Bacteria	*Bacteroidetes*	*Sphingobacteriia*	*Sphingobacteriales*	*Sphingobacteriaceae*	0,000	0,000	0,011	0,000	0,003	0,000	0,000	0,000	0,000	0,000	0,000	0,000	0,000	0,000	0,000	0,000	0,000	0,000	0,000	0,000	0,000	0,010	0,002
Bacteria	*Proteobacteria*	*Alphaproteobacteria*	*Sphingomonadales*	*Sphingomonadaceae*	0,000	0,000	0,000	0,000	0,000	0,000	0,000	0,000	0,000	0,000	0,000	0,000	0,000	0,000	0,000	0,000	0,000	0,000	0,000	0,000	0,000	0,000	0,000
Bacteria	*Spirochaetes*	*Spirochaetes*	*Spirochaetales*	*Spirochaetaceae*	0,716	0,272	0,845	1,000	0,708	0,000	0,000	0,285	0,016	0,066	0,073	0,792	0,446	0,163	0,699	0,908	0,602	0,028	0,034	0,000	0,119	0,269	0,090
Bacteria	*Firmicutes*	*Bacilli*	*Lactobacillales*	*Streptococcaceae*	0,258	0,000	0,000	0,063	0,080	0,148	0,082	0,214	0,049	0,175	0,134	0,000	0,143	0,040	0,000	0,000	0,037	0,000	0,149	0,000	0,215	0,035	0,080
Bacteria	*Proteobacteria*	*Gammaproteobacteria*	*Aeromonadales*	*Succinivibrionaceae*	0,156	0,072	0,000	0,000	0,057	0,000	0,092	0,251	0,000	0,361	0,141	0,000	0,000	0,069	0,361	0,000	0,086	0,209	0,149	0,000	0,000	0,024	0,076
Bacteria	*Synergistetes*	*Synergistia*	*Synergistales*	*Synergistaceae*	0,000	0,000	0,000	0,000	0,000	0,000	0,000	0,000	0,000	0,000	0,000	0,000	0,000	0,000	0,000	0,000	0,000	0,000	0,000	0,000	0,000	0,000	0,000
Bacteria	*Firmicutes*	*Bacilli*	*Turicibacterales*	*Turicibacteraceae*	0,000	0,000	0,000	0,000	0,000	0,000	0,000	0,000	0,000	0,000	0,000	0,000	0,000	0,000	0,000	0,000	0,000	0,000	0,000	0,000	0,000	0,000	0,000
Bacteria	*Tenericutes*	*Mollicutes*	*Acholeplasmatales*	*Unclassified*	0,000	0,000	0,000	0,000	0,000	0,000	0,000	0,000	0,000	0,051	0,010	0,000	0,000	0,000	0,000	0,000	0,000	0,000	0,000	0,000	0,000	0,000	0,000
Bacteria	*Tenericutes*	*Mollicutes*	*Acholeplasmatales*	*Unclassified*	0,000	0,000	0,000	0,810	0,202	0,000	0,000	0,000	0,000	0,000	0,000	0,000	0,000	0,000	0,000	0,000	0,000	0,000	0,000	0,000	0,000	0,000	0,000
Bacteria	*Actinobacteria*	*Actinobacteria*	*Actinomycetales*	*Unclassified*	0,000	0,112	0,220	0,169	0,125	0,000	0,000	0,217	0,000	0,204	0,084	0,000	0,000	0,179	0,000	0,000	0,036	0,000	0,000	0,000	0,000	0,103	0,021
Bacteria	*Firmicutes*	*Bacilli*	*Bacillales*	*Unclassified*	0,439	0,526	0,233	0,000	0,300	0,000	0,638	0,442	0,430	0,000	0,302	0,000	0,242	0,000	0,127	0,000	0,074	0,699	0,330	0,000	0,342	0,327	0,340
Bacteria	*Bacteroidetes*	*Bacteroidia*	*Bacteroidales*	*Unclassified*	3,477	4,094	5,910	11,952	6,358	7,049	4,657	3,875	6,064	3,738	5,077	7,707	4,410	9,578	14,987	11,333	9,603	1,830	5,158	3,676	3,387	2,761	3,362
Bacteria	*Bacteroidetes*	*Bacteroidia*	*Bacteroidales*	*Unclassified*	2,917	0,663	4,844	3,914	3,085	3,528	0,075	1,395	0,966	0,120	1,217	0,927	0,611	1,341	1,922	3,260	1,612	0,264	0,482	0,000	2,792	1,558	1,019
Bacteria	*TM7*	*TM7-3*	*Blgi18*	*Unclassified*	0,000	0,000	0,000	0,000	0,000	0,000	0,000	0,000	0,000	0,000	0,000	0,000	0,000	0,000	0,000	0,000	0,000	0,000	0,000	0,000	0,000	0,000	0,000
Bacteria	*Proteobacteria*	*Betaproteobacteria*	*Burkholderiales*	*Unclassified*	0,000	0,000	0,189	0,000	0,047	0,000	0,000	0,130	0,435	0,026	0,118	0,000	0,000	0,095	0,000	0,467	0,113	0,000	0,000	0,000	0,070	0,000	0,014
Bacteria	*Chlamydiae*	*Chlamydiia*	*Chlamydiales*	*Unclassified*	0,000	0,000	0,000	0,000	0,000	0,000	0,000	0,000	0,000	0,000	0,000	0,000	0,000	0,000	0,000	0,000	0,000	0,000	0,000	0,000	0,000	0,015	0,003
Bacteria	*Firmicutes*	*Clostridia*	*Clostridiales*	*Unclassified*	6,992	9,502	10,509	7,684	8,672	10,683	1,476	11,592	10,736	8,029	8,503	6,009	11,666	9,651	3,711	9,081	8,024	4,875	7,345	2,010	8,334	9,647	6,442
Bacteria	*Firmicutes*	*Clostridia*	*Clostridiales*	*Unclassified*	3,914	3,441	0,327	1,666	2,337	4,846	0,350	2,788	2,403	4,189	2,915	0,721	3,131	2,174	2,233	1,953	2,042	3,105	3,427	1,331	2,956	1,653	2,495
Bacteria	*TM7*	*TM7-3*	*CW040*	*Unclassified*	0,000	0,000	0,000	0,000	0,000	0,000	0,000	0,000	0,000	0,000	0,000	0,000	0,000	0,000	0,000	0,000	0,000	0,000	0,000	0,000	0,000	0,000	0,000
Bacteria	*Firmicutes*	*Bacilli*	*Lactobacillales*	*Unclassified*	0,000	0,000	0,000	0,000	0,000	0,000	0,000	0,000	0,003	0,000	0,001	0,000	0,000	0,000	0,000	0,000	0,000	0,000	0,000	0,000	0,000	0,000	0,000
Bacteria	*Proteobacteria*	*Alphaproteobacteria*	*RF32*	*Unclassified*	0,000	0,000	0,000	0,000	0,000	0,000	0,000	0,000	0,000	0,000	0,000	0,000	0,000	0,000	0,000	0,000	0,000	0,000	0,000	0,000	0,000	0,000	0,000
Bacteria	*Proteobacteria*	*Alphaproteobacteria*	*Rhizobiales*	*Unclassified*	0,000	0,000	0,000	0,008	0,002	0,000	0,000	0,000	0,000	0,000	0,000	0,000	0,000	0,000	0,000	0,000	0,000	0,000	0,000	0,000	0,000	0,000	0,000
Bacteria	*Proteobacteria*	*Alphaproteobacteria*	*Rhodospirillales*	*Unclassified*	0,226	0,147	0,901	0,207	0,370	0,000	0,000	0,000	0,000	0,000	0,000	0,507	0,000	0,118	0,000	0,000	0,125	0,000	0,000	0,000	1,279	0,185	0,293
Bacteria	*Synergistetes*	*Synergistia*	*Synergistales*	*Unclassified*	0,000	0,000	0,000	0,000	0,000	0,000	0,000	0,000	0,000	0,000	0,000	0,000	0,000	0,000	0,000	0,000	0,000	0,000	0,000	0,000	0,000	0,000	0,000
Bacteria	*Proteobacteria*	*Betaproteobacteria*	*Tremblayales*	*Unclassified*	0,000	0,000	0,000	0,000	0,000	0,000	0,000	0,000	0,000	0,000	0,000	0,000	0,000	0,000	0,000	0,000	0,000	0,000	0,000	0,000	0,170	0,000	0,034
Bacteria	*Cyanobacteria*	*4C0d-2*	*YS2*	*Unclassified*	0,147	0,530	0,191	0,198	0,267	0,000	0,000	0,417	0,000	0,000	0,083	0,021	0,033	0,100	0,000	0,100	0,051	0,000	0,000	0,000	0,732	0,063	0,159
Unassigned	*Unclassified*	*Unclassified*	*Unclassified*	*Unclassified*	0,000	0,000	0,000	0,000	0,000	0,000	0,000	0,000	0,000	0,000	0,000	0,000	0,000	0,000	0,000	0,000	0,000	0,000	0,000	0,000	0,000	0,000	0,000
Bacteria	*Unclassified*	*Unclassified*	*Unclassified*	*Unclassified*	7,698	7,910	13,470	13,466	10,636	10,774	0,772	4,938	28,965	10,226	11,135	11,497	8,375	8,153	5,770	8,934	8,546	12,222	7,818	11,492	22,112	25,472	15,823
Bacteria	*Bacteroidetes*	*Unclassified*	*Unclassified*	*Unclassified*	1,653	1,440	3,054	1,037	1,796	0,481	0,007	0,559	0,848	0,255	0,430	2,092	1,005	1,277	3,397	0,945	1,743	0,000	0,425	0,000	2,431	0,477	0,667
Bacteria	*Firmicutes*	*Unclassified*	*Unclassified*	*Unclassified*	3,008	2,785	3,830	2,590	3,053	2,555	0,959	2,229	3,071	2,911	2,345	3,170	3,365	2,123	1,638	2,892	2,638	3,075	2,735	1,202	2,928	2,612	2,511
Bacteria	*Unclassified*	*Unclassified*	*Unclassified*	*Unclassified*	0,152	0,110	0,420	0,333	0,254	0,000	0,000	0,210	0,057	0,295	0,112	0,021	0,544	0,136	0,154	0,000	0,171	0,181	0,772	0,000	0,139	0,077	0,234
Bacteria	*Spirochaetes*	*Spirochaetes*	*Unclassified*	*Unclassified*	0,904	0,341	0,601	1,029	0,719	0,541	0,000	0,228	0,348	0,000	0,223	1,459	1,200	1,350	1,237	1,927	1,435	0,000	0,023	0,000	0,000	0,434	0,091
Bacteria	*Firmicutes*	*Clostridia*	*Unclassified*	*Unclassified*	0,317	0,845	0,890	1,143	0,799	1,193	0,262	0,235	0,033	0,164	0,377	0,981	0,368	0,746	0,441	1,207	0,749	0,000	1,085	1,013	0,275	0,343	0,543
Bacteria	*Proteobacteria*	*Alphaproteobacteria*	*Unclassified*	*Unclassified*	0,000	0,000	0,000	0,000	0,000	0,000	0,000	0,000	0,961	0,000	0,192	1,484	0,052	0,000	0,140	0,000	0,335	0,035	0,066	0,000	0,771	0,276	0,230
Bacteria	*Tenericutes*	*Mollicutes*	*Unclassified*	*Unclassified*	0,000	0,000	0,191	0,000	0,048	0,000	0,000	0,000	0,000	0,044	0,009	1,073	0,000	0,000	0,000	0,000	0,215	0,000	0,000	0,000	0,000	0,000	0,000
Bacteria	*Proteobacteria*	*Unclassified*	*Unclassified*	*Unclassified*	0,362	0,202	0,283	0,388	0,309	0,000	0,000	0,000	0,033	0,000	0,007	0,050	0,000	0,000	0,000	0,000	0,010	0,000	0,000	0,000	0,209	0,000	0,042
Bacteria	*Cyanobacteria*	*Unclassified*	*Unclassified*	*Unclassified*	0,136	0,088	0,134	0,000	0,090	0,000	0,121	0,134	0,501	0,197	0,191	0,000	0,102	0,175	0,000	0,000	0,055	0,128	0,218	0,000	0,203	0,074	0,125
Bacteria	*Firmicutes*	*Bacilli*	*Unclassified*	*Unclassified*	0,000	0,037	0,000	0,000	0,009	0,000	0,000	0,000	0,000	0,015	0,003	0,000	0,000	0,000	0,000	0,000	0,000	0,000	0,020	0,000	0,000	0,000	0,004
Bacteria	*Lentisphaerae*	*Unclassified*	*Unclassified*	*Unclassified*	0,159	0,131	0,000	0,000	0,072	0,000	0,000	0,253	0,000	0,058	0,062	0,000	0,000	0,116	0,000	0,000	0,023	0,000	0,227	0,000	0,068	0,000	0,059
Bacteria	*OD1*	*Unclassified*	*Unclassified*	*Unclassified*	0,000	0,000	0,000	0,000	0,000	0,000	0,000	0,000	0,000	0,000	0,000	0,000	0,000	0,000	0,000	0,000	0,000	0,000	0,000	0,000	0,000	0,000	0,000
Bacteria	*Proteobacteria*	*Betaproteobacteria*	*Unclassified*	*Unclassified*	0,000	0,016	0,000	0,000	0,004	0,000	0,000	0,018	0,000	0,000	0,004	0,000	0,123	0,001	0,147	0,000	0,054	0,000	0,000	0,000	0,008	0,000	0,002
Bacteria	*Proteobacteria*	*Deltaproteobacteria*	*Unclassified*	*Unclassified*	0,068	0,000	0,000	0,000	0,017	0,000	0,000	0,000	0,000	0,000	0,000	0,000	0,000	0,000	0,000	0,000	0,000	0,000	0,000	0,000	0,000	0,000	0,000
Bacteria	*Proteobacteria*	*Gammaproteobacteria*	*Unclassified*	*Unclassified*	0,163	0,000	0,000	0,000	0,041	0,000	0,000	0,000	0,000	0,000	0,000	0,000	0,000	0,000	0,000	0,000	0,000	0,000	0,347	0,000	0,000	0,244	0,118
Bacteria	*Tenericutes*		*Unclassified*	*Unclassified*	0,000	0,000	0,000	0,000	0,000	0,000	0,000	0,000	0,000	0,000	0,000	0,000	0,000	0,000	0,000	0,000	0,000	0,000	0,000	0,000	0,000	0,000	0,000
Bacteria	*Firmicutes*	*Clostridia*	*Clostridiales*	*Veillonellaceae*	2,013	2,008	1,290	2,826	2,034	1,628	2,494	2,150	0,917	3,166	2,071	2,034	2,273	1,220	0,879	1,160	1,513	3,010	1,561	0,000	1,007	1,751	1,466
Bacteria	*Verrucomicrobia*	*Verrucomicrobiae*	*Verrucomicrobiales*	*Verrucomicrobiaceae*	0,000	0,043	0,000	0,000	0,011	0,000	0,000	0,000	0,000	0,000	0,000	0,000	0,000	0,000	0,000	0,000	0,000	0,000	0,000	0,000	0,000	0,034	0,007
Bacteria	*Lentisphaerae*	*[Lentisphaeria]*	*Victivallales*	*Victivallaceae*	0,199	0,000	0,104	0,000	0,076	1,049	0,000	0,253	0,213	0,186	0,340	0,084	0,210	0,033	0,000	0,110	0,087	0,000	0,356	0,000	0,086	0,000	0,088
Bacteria	*Verrucomicrobia*	*Verruco-5*	*WCHB1-41*	*WCHB1-25*	0,000	0,000	0,035	0,000	0,009	0,000	0,000	0,000	0,000	0,000	0,000	0,000	0,000	0,000	0,000	0,000	0,000	0,000	0,000	0,000	0,000	0,000	0,000
Bacteria	*Proteobacteria*	*Gammaproteobacteria*	*Xanthomonadales*	*Xanthomonadaceae*	0,000	0,000	0,000	0,000	0,000	0,000	0,000	0,000	0,000	0,000	0,000	0,000	0,000	0,000	0,000	0,000	0,000	0,000	0,000	0,000	0,000	0,000	0,000

Supplementary Table S2. Relative abundance (%) of taxa assigned at different levels (Phyla, Family and Genera) for each animal sample and mean relative abundance (%) for each group of animals (cont.).

						PF_15dpft					WF_15dpft						AWF_15dpft						PBS_15dpft					
Kingdom	Phyla	Class	Order	Family	Genus	16	18	22	24	AVERAGE	25	28	29	30	36	AVERAGE	37	41	43	44	46	AVERAGE	1	3	4	9	11	AVERAGE
Bacteria	Firmicutes	Erysipelotrichi	Erysipelotrichales	Erysipelotrichaceae	[Eubacterium]	0,349	0,025	0,016	0,000	0,098	0,087	0,000	0,150	0,066	0,448	0,150	0,000	0,134	0,103	0,100	0,126	0,093	0,000	0,066	0,000	0,000	0,058	0,025
Bacteria	Bacteroidetes	Bacteroidia	Bacteroidales	[Paraprevotellaceae]	[Prevotella]	1,431	0,626	1,203	0,430	0,923	0,307	0,389	0,661	1,221	0,077	0,531	1,287	0,351	0,830	1,461	1,186	1,023	0,699	0,000	0,000	3,377	0,811	0,977
Bacteria	Tenericutes	Mollicutes	Acholeplasmatales	Acholeplasmataceae	Acholeplasma	0,000	0,086	0,042	0,540	0,167	0,053	0,000	0,000	0,000	0,000	0,011	0,273	0,171	0,000	0,114	0,115	0,135	0,000	0,000	0,000	0,086	0,010	0,019
Bacteria	Proteobacteria	Gammaproteobacteria	Pasteurellales	Pasteurellaceae	Actinobacillus	0,036	0,061	0,027	0,114	0,060	0,000	0,118	0,048	0,000	0,040	0,041	0,075	0,000	0,093	0,000	0,000	0,034	0,000	0,000	0,000	0,000	0,042	0,008
Bacteria	Verrucomicrobia	Verrucomicrobiae	Verrucomicrobiales	Verrucomicrobiaceae	Akkermansia	0,000	0,043	0,000	0,000	0,011	0,000	0,000	0,000	0,000	0,000	0,000	0,000	0,000	0,000	0,000	0,000	0,000	0,000	0,000	0,000	0,000	0,000	0,000
Bacteria	Bacteroidetes	Bacteroidia	Bacteroidales	Rikenellaceae	Alistipes	0,041	0,000	0,000	0,000	0,010	0,000	0,056	0,196	0,000	0,087	0,068	0,000	0,000	0,042	0,498	0,079	0,124	0,000	0,029	0,000	0,000	0,027	0,011
Bacteria	Proteobacteria	Gammaproteobacteria	Pseudomonadales	Moraxellaceae	Alkanindiges	0,000	0,000	0,000	0,000	0,000	0,000	0,000	0,000	0,000	0,000	0,000	0,000	0,000	0,000	0,000	0,000	0,000	0,000	0,000	0,000	0,000	0,000	0,000
Bacteria	Proteobacteria	Gammaproteobacteria	Aeromonadales	Succinivibrionaceae	Anaerobiospirillum	0,156	0,072	0,000	0,000	0,057	0,000	0,092	0,251	0,000	0,117	0,092	0,000	0,000	0,069	0,361	0,000	0,086	0,209	0,149	0,000	0,000	0,024	0,076
Bacteria	Firmicutes	Clostridia	Clostridiales	Eubacteriaceae	Anaerofustis	0,000	0,000	0,000	0,000	0,000	0,000	0,000	0,000	0,000	0,000	0,000	0,000	0,000	0,037	0,000	0,063	0,020	0,000	0,000	0,000	0,000	0,060	0,012
Bacteria	Tenericutes	Mollicutes	Anaeroplasmatales	Anaeroplasmataceae	Anaeroplasma	0,000	0,000	0,000	0,059	0,015	0,000	0,000	0,000	0,000	0,000	0,000	0,000	0,119	0,000	0,000	0,000	0,024	0,000	0,000	0,000	0,000	0,000	0,000
Bacteria	Firmicutes	Erysipelotrichi	Erysipelotrichales	Erysipelotrichaceae	Anaerorhabdus	0,070	0,000	0,187	0,059	0,079	0,000	0,000	0,050	0,000	0,000	0,010	0,038	0,240	0,076	0,378	0,472	0,241	0,045	0,000	0,000	0,599	0,000	0,129
Bacteria	Firmicutes	Clostridia	Clostridiales	Veillonellaceae	Anaerosinus	0,000	0,000	0,000	0,000	0,000	0,000	0,000	0,000	0,000	0,000	0,000	0,000	0,000	0,000	0,000	0,000	0,000	0,000	0,000	0,000	0,000	0,000	0,000
Bacteria	Firmicutes	Clostridia	Clostridiales	Ruminococcaceae	Anaerotruncus	0,000	0,000	0,000	0,000	0,000	0,000	0,000	0,000	0,000	0,000	0,000	0,000	0,000	0,000	0,000	0,000	0,000	0,000	0,000	0,000	0,000	0,000	0,000
Bacteria	Actinobacteria	Actinobacteria	Actinomycetales	Micrococcaceae	Arthrobacter	0,000	0,000	0,000	0,000	0,000	0,000	0,000	0,000	0,000	0,000	0,000	0,000	0,000	0,000	0,000	0,000	0,000	0,000	0,000	0,000	0,000	0,000	0,000
Bacteria	Cyanobacteria	Oscillatoriophycideae	Oscillatoriales	Phormidiaceae	Arthrospira	0,000	0,000	0,000	0,000	0,000	0,000	0,000	0,000	0,000	0,000	0,000	0,000	0,000	0,000	0,000	0,000	0,000	0,000	0,000	0,000	0,000	0,000	0,000
Bacteria	Firmicutes	Erysipelotrichi	Erysipelotrichales	Erysipelotrichaceae	Asteroleplasma	1,522	1,413	0,438	0,084	0,864	0,594	4,817	0,878	1,497	1,902	1,938	0,273	0,769	2,523	0,435	0,467	0,893	1,300	1,814	1,494	0,094	1,840	1,309
Bacteria	Firmicutes	Bacilli	Bacillales	Bacillaceae	Bacillus	0,000	0,000	0,000	0,000	0,000	0,000	0,000	0,000	0,000	0,000	0,000	0,000	0,000	0,000	0,000	0,000	0,000	0,008	0,000	0,000	0,025	0,000	0,006
Bacteria	Bacteroidetes	Bacteroidia	Bacteroidales	Bacteroidaceae	Bacteroides	0,000	0,000	0,000	0,000	0,000	0,000	0,000	0,089	0,052	0,128	0,054	0,000	0,020	0,000	0,000	0,000	0,004	0,096	0,112	0,000	0,103	0,024	0,067
Bacteria	Firmicutes	Clostridia	Clostridiales	Ruminococcaceae	Bacteroides	0,068	0,000	0,000	0,000	0,017	0,000	0,000	0,251	0,000	0,058	0,062	0,000	0,191	0,000	0,000	0,000	0,038	0,000	0,000	0,000	0,129	0,068	0,039
Bacteria	Bacteroidetes	Bacteroidia	Bacteroidales	[Barnesiellaceae]	Barnesiella	0,303	0,546	0,000	1,088	0,484	0,572	0,507	0,030	0,000	0,368	0,295	0,805	0,894	0,655	2,898	0,835	1,217	0,000	0,000	0,000	0,000	0,000	0,000
Bacteria	Bacteroidetes	Flavobacteriia	Flavobacteriales	[Weeksellaceae]	Bergeyella	0,000	0,000	0,000	0,000	0,000	0,000	0,000	0,000	0,000	0,000	0,000	0,000	0,000	0,000	0,000	0,000	0,000	0,068	0,000	0,000	0,000	0,000	0,014
Bacteria	Actinobacteria	Actinobacteria	Bifidobacteriales	Bifidobacteriaceae	Bifidobacterium	0,000	0,000	0,000	0,000	0,000	0,000	0,000	0,000	0,000	0,000	0,000	0,000	0,000	0,000	0,000	0,000	0,000	0,000	0,000	0,000	0,000	0,000	0,000
Bacteria	Spirochaetes	[Brachyspirae]	[Brachyspirales]	Brachyspiraceae	Brachyspira	0,000	0,000	0,000	0,000	0,000	0,000	0,000	0,000	0,000	0,000	0,000	0,000	0,000	0,000	0,000	0,000	0,000	0,000	0,000	0,000	0,000	0,000	0,000
Bacteria	Proteobacteria	Alphaproteobacteria	Caulobacterales	Caulobacteraceae	Brevundimonas	0,000	0,000	0,000	0,000	0,000	0,000	0,000	0,000	0,000	0,000	0,000	0,000	0,000	0,000	0,017	0,000	0,003	0,000	0,000	0,000	0,000	0,000	0,000
Bacteria	Firmicutes	Clostridia	Clostridiales	Ruminococcaceae	Butyricicoccus	0,464	0,524	1,099	0,177	0,566	0,617	0,376	0,994	0,389	0,350	0,545	0,356	0,613	0,807	0,000	0,257	0,407	1,260	1,266	0,000	0,820	0,314	0,732
Bacteria	Firmicutes	Clostridia	Clostridiales	Lachnospiraceae	Butyrivibrio	0,000	0,000	0,000	0,000	0,000	0,000	0,000	0,000	0,000	0,000	0,000	0,000	0,000	0,000	0,000	0,000	0,000	0,000	0,000	0,000	0,000	0,000	0,000
Bacteria	Proteobacteria	Epsilonproteobacteria	Campylobacterales	Campylobacteraceae	Campylobacter	0,131	0,047	0,067	0,245	0,123	0,000	0,000	0,367	0,131	0,692	0,238	0,084	0,000	0,224	0,000	0,000	0,062	0,123	0,077	0,000	0,568	0,032	0,160
Bacteria	Firmicutes	Erysipelotrichi	Erysipelotrichales	Erysipelotrichaceae	Catenibacterium	0,000	0,000	0,000	0,000	0,000	0,117	0,000	0,000	0,000	0,000	0,023	0,134	0,050	0,000	0,000	0,000	0,037	0,000	0,000	0,000	0,000	0,123	0,025
Bacteria	Chlamydiae	Chlamydiia	Chlamydiales	Chlamydiaceae	Chlamydia	0,000	0,168	0,000	0,000	0,042	0,000	0,383	0,000	0,000	0,521	0,181	0,583	0,106	0,145	0,204	1,475	0,502	0,221	0,000	0,000	0,000	0,102	0,065
Bacteria	Firmicutes	Clostridia	Clostridiales	Christensenellaceae	Christensenella	0,000	0,000	0,000	0,000	0,000	0,000	0,000	0,000	0,000	0,000	0,000	0,000	0,000	0,000	0,000	0,084	0,017	0,000	0,000	0,000	0,000	0,000	0,000
Bacteria	Firmicutes	Clostridia	Clostridiales	Clostridiaceae	Clostridium	1,964	11,281	0,899	1,396	3,885	3,759	0,471	2,113	0,290	2,645	1,856	3,686	3,455	2,245	3,818	1,512	2,943	5,579	2,003	8,701	0,000	1,069	3,470
Bacteria	Firmicutes	Clostridia	Clostridiales	Lachnospiraceae	Clostridium	0,299	0,450	0,044	0,139	0,233	0,000	0,121	0,164	0,000	1,158	0,289	0,000	0,243	0,545	0,398	0,068	0,251	0,385	0,342	0,000	0,123	0,158	0,201
Bacteria	Firmicutes	Clostridia	Clostridiales	Peptostreptococcaceae	Clostridium	0,503	0,685	0,000	0,198	0,346	0,000	0,000	0,000	0,000	0,000	0,000	0,616	0,632	0,474	0,986	0,000	0,542	0,453	0,000	0,000	0,000	0,114	0,113
Bacteria	Firmicutes	Clostridia	Clostridiales	Ruminococcaceae	Clostridium	3,875	3,370	7,884	2,619	4,437	3,328	0,792	2,524	3,002	3,173	2,564	4,851	5,632	3,148	3,844	3,900	4,275	6,205	5,029	2,130	3,963	4,330	4,331
Bacteria	Firmicutes	Erysipelotrichi	Erysipelotrichales	Erysipelotrichaceae	Clostridium	0,000	0,000	0,064	0,000	0,016	0,000	0,000	0,000	0,000	0,000	0,000	0,000	0,000	0,000	0,000	0,000	0,000	0,000	0,000	0,000	0,000	0,000	0,000
Bacteria	Firmicutes	Clostridia	Clostridiales	Lachnospiraceae	Coprococcus	0,000	0,000	0,000	0,000	0,000	0,000	0,000	0,000	0,000	0,000	0,000	0,000	0,000	0,000	0,000	0,000	0,000	0,000	0,000	0,000	0,000	0,000	0,000
Archaea	Euryarchaeota	Thermoplasmata	E2	[Methanomassiliicoccaceae]	Coxiella	0,000	0,000	0,000	0,000	0,000	0,000	0,000	0,000	0,000	0,058	0,012	0,046	0,000	0,000	0,000	0,000	0,009	0,000	0,000	0,000	0,000	0,024	0,005
Bacteria	Bacteroidetes	Bacteroidia	Bacteroidales	Marinilabiaceae	Cytophaga	0,000	0,000	0,000	0,000	0,000	0,000	0,000	0,000	0,000	0,000	0,000	0,000	0,000	0,000	0,000	0,000	0,000	0,000	0,000	0,000	0,000	0,000	0,000
Bacteria	Bacteroidetes	Sphingobacteriia	Sphingobacteriales	Sphingobacteriaceae	Cytophagales	0,000	0,000	0,000	0,000	0,000	0,000	0,000	0,000	0,000	0,000	0,000	0,000	0,000	0,000	0,000	0,000	0,000	0,000	0,000	0,000	0,000	0,010	0,002
Bacteria	Firmicutes	Clostridia	Clostridiales	Lachnospiraceae	Defluviitalea	0,000	0,000	0,045	0,249	0,074	0,000	0,000	0,107	0,000	0,641	0,150	0,000	0,255	0,055	0,023	0,000	0,067	0,277	0,118	0,000	0,312	0,056	0,152
Bacteria	Proteobacteria	Betaproteobacteria	Rhodocyclales	Rhodocyclaceae	Denitromonas	0,000	0,000	0,000	0,000	0,000	0,000	0,000	0,000	0,000	0,000	0,000	0,000	0,000	0,000	0,000	0,000	0,000	0,000	0,000	0,000	0,000	0,000	0,000
Bacteria	Proteobacteria	Deltaproteobacteria	Desulfovibrionales	Desulfovibrionaceae	Desulfovibrio	0,267	0,084	0,116	0,000	0,117	0,000	0,157	0,036	0,047	0,313	0,111	0,046	0,061	0,048	0,084	0,084	0,065	0,103	0,000	0,000	0,000	0,045	0,030
Bacteria	Firmicutes	Clostridia	Clostridiales	Lachnospiraceae	Dorea	0,000	0,000	0,000	0,000	0,000	0,000	0,000	0,000	0,000	0,222	0,044	0,000	0,000	0,000	0,000	0,000	0,000	0,000	0,000	0,000	0,000	0,106	0,021
Bacteria	Bacteroidetes	Flavobacteriia	Flavobacteriales	[Weeksellaceae]	Elizabethkingia	0,000	0,000	0,000	0,000	0,000	0,000	0,000	0,000	0,000	0,000	0,000	0,000	0,000	0,000	0,000	0,000	0,000	0,000	0,000	0,000	0,047	0,000	0,009
Bacteria	Verrucomicrobia	[Spartobacteria]	[Chthoniobacterales]	[Chthoniobacteraceae]	Ellin506	0,000	0,000	0,000	0,000	0,000	0,000	0,000	0,000	0,000	0,000	0,000	0,000	0,000	0,000	0,000	0,000	0,000	0,000	0,034	0,000	0,000	0,000	0,007
Bacteria	Elusimicrobia	Elusimicrobia	Elusimicrobiales	Elusimicrobiaceae	Elusimicrobium	0,000	0,029	0,031	0,000	0,015	0,011	0,000	0,000	0,071	0,120	0,041	0,000	0,000	0,000	0,007	0,315	0,064	0,000	0,000	0,000	0,000	0,035	0,007
Bacteria	Proteobacteria	Gammaproteobacteria	Pseudomonadales	Moraxellaceae	Enhydrobacter	0,000	0,000	0,000	0,000	0,000	0,000	0,000	0,000	0,000	0,000	0,000	0,000	0,000	0,000	0,017	0,000	0,003	0,000	0,000	0,000	0,000	0,000	0,000
Bacteria	Actinobacteria	Coriobacteriia	Coriobacteriales	Coriobacteriaceae	Enterococcus	0,000	0,000	0,000	0,000	0,000	0,000	0,000	0,000	0,000	0,000	0,000	0,000	0,000	0,000	0,000	0,000	0,000	0,000	0,000	0,000	0,000	0,000	0,000
Bacteria	Firmicutes	Clostridia	Clostridiales	Ruminococcaceae	Faecalibacterium	1,671	1,544	0,667	1,708	1,398	1,302	0,448	1,133	1,387	1,876	1,229	1,455	0,775	0,570	0,294	0,861	0,791	1,514	1,019	0,000	0,707	0,914	0,831
Bacteria	Fibrobacteres	Fibrobacteria	Fibrobacterales	Fibrobacteraceae	Fibrobacter	0,000	0,029	0,000	0,101	0,033	0,000	0,098	0,000	0,000	0,000	0,020	0,000	0,279	0,064	0,000	0,152	0,099	0,000	0,000	0,000	0,000	0,189	0,038
Bacteria	Firmicutes	Clostridia	Clostridiales	[Tissierellaceae]	Finegoldia	0,000	0,000	0,000	0,000	0,000	0,000	0,000	0,000	0,000	0,000	0,000	0,000	0,000	0,000	0,000	0,000	0,000	0,000	0,000	0,000	0,000	0,000	0,000
Bacteria	Firmicutes	Clostridia	Clostridiales	Ruminococcaceae	Gemmiger	0,360	0,380	0,214	0,000	0,239	0,000	0,749	0,358	0,588	0,466	0,432	0,184	0,710	1,109	0,294	0,000	0,459	1,398	0,390	0,928	1,085	0,319	0,824
Bacteria	Tenericutes	Mollicutes	Anaeroplasmatales	Anaeroplasmataceae	gut	0,000	0,000	0,000	0,000	0,000	0,000	0,000	0,000	0,000	0,000	0,000	0,000	0,000	0,000	0,000	0,000	0,000	0,000	0,000	0,000	0,000	0,000	0,000
Bacteria	Firmicutes	Erysipelotrichi	Erysipelotrichales	Erysipelotrichaceae	gut	1,803	3,470	1,145	1,898	2,079	3,169	8,296	3,119	2,425	3,639	4,129	1,187	1,907	4,618	1,849	2,583	2,428	4,254	7,035	2,534	0,752	1,866	3,288
Bacteria	Firmicutes	Clostridia	Clostridiales	[Tissierellaceae]	GW-34	0,000	0,000	0,000	0,000	0,000	0,000	0,000	0,000	0,000	0,000	0,000	0,000	0,000	0,000	0,000	0,000	0,000	0,000	0,000	0,000	0,000	0,000	0,000
Bacteria	Proteobacteria	Epsilonproteobacteria	Campylobacterales	Helicobacteraceae	Helicobacter	0,000	0,000	0,000	0,139	0,035	0,106	0,000	0,000	0,183	0,000	0,058	0,159	0,000	0,210	0,274	0,000	0,129	0,111	0,049	0,000	0,324	0,000	0,097
Bacteria	Proteobacteria	Betaproteobacteria	Burkholderiales	Oxalobacteraceae	Herbaspirillum	0,000	0,000	0,000	0,000	0,000	0,000	0,000	0,000	0,128	0,000	0,026	0,042	0,000	0,000	0,067	0,215	0,065	0,000	0,000	0,000	0,000	0,000	0,000
Bacteria	Firmicutes	Clostridia	Clostridiales	Lachnospiraceae	Hespellia	0,097	0,086	0,000	0,000	0,046	0,000	0,000	0,000	0,000	0,353	0,071	0,000	0,039	0,000	0,000	0,231	0,054	0,000	0,057	0,000	0,000	0,184	0,048
Bacteria	Firmicutes	Clostridia	Clostridiales	Lachnospiraceae	Lachnospira	0,000	0,000	0,000	0,000	0,000	0,000	0,000	0,000	0,000	0,000	0,000	0,000	0,000	0,000	0,000	0,000	0,000	0,000	0,000	0,000	0,000	0,000	0,000
Bacteria	Firmicutes	Bacilli	Lactobacillales	Lactobacillaceae	Lactobacillus	0,000	0,000	0,420	0,000	0,105	0,000	0,052	0,000	0,060	0,000	0,023	0,000	0,000	0,010	0,000	0,000	0,002	0,023	0,000	0,000	0,000	0,044	0,013
Bacteria	Firmicutes	Clostridia	Clostridiales	Lachnospiraceae	Lactonifactor	0,000	0,000	0,000	0,000	0,000	0,182	0,000	0,000	0,000	0,000	0,036	0,331	0,000	0,164	0,000	0,803	0,260	0,000	0,000	0,000	0,441	0,263	0,141
Bacteria	Proteobacteria	Betaproteobacteria	Burkholderiales	Comamonadaceae	Limnobacter	0,000	0,025	0,000	0,000	0,006	0,000	0,000	0,041	0,000	0,000	0,008	0,000	0,000	0,000	0,053	0,000	0,011	0,043	0,000	0,000	0,000	0,000	0,009
Bacteria	Bacteroidetes	Bacteroidia	Bacteroidales	Porphyromonadaceae	Macellibacteroides	0,000	0,000	0,000	0,000	0,000	0,000	0,000	0,000	0,000	0,000	0,000	0,000	0,000	0,000	0,000	0,000	0,000	0,000	0,000	0,000	0,000	0,000	0,000
Bacteria	Firmicutes	Clostridia	Clostridiales	Lachnospiraceae	Marvinbryantia	0,000	0,000	0,000	0,000	0,000	0,000	0,000	0,000	0,000	0,000	0,000	0,000	0,000	0,000	0,000	0,000	0,000	0,000	0,000	0,000	0,000	0,000	0,000
Archaea	Euryarchaeota	Methanobacteria	Methanobacteriales	Methanobacteriaceae	Methanobrevibacter	0,000	0,000	0,000	0,000	0,000	0,000	0,000	0,000	0,000	0,000	0,000	0,092	0,000	0,000	0,000	0,168	0,052	0,000	0,000	0,000	0,000	0,000	0,000
Archaea	Euryarchaeota	Methanobacteria	Methanobacteriales	Methanobacteriaceae	Methanosphaera	0,000	0,000	0,000	0,034	0,008	0,000	0,000	0,000	0,000	0,000	0,000	0,021	0,000	0,000	0,000	0,000	0,004	0,000	0,000	0,000	0,000	0,000	0,000
Bacteria	Actinobacteria	Actinobacteria	Actinomycetales	Gordoniaceae	Millisia	0,000	0,000	0,000	0,000	0,000	0,000	0,000	0,000	0,000	0,000	0,000	0,000	0,000	0,000	0,000	0,000	0,000	0,000	0,000	0,000	0,000	0,000	0,000
Bacteria	Proteobacteria	Gammaproteobacteria	Pseudomonadales	Moraxellaceae	Moraxella	0,000	0,000	0,000	0,000	0,000	0,000	0,000	0,000	0,000	0,186	0,037	0,000	0,000	0,022	0,000	0,000	0,004	0,000	0,000	0,000	0,000	0,000	0,000
Bacteria	Deferribacteres	Deferribacteres	Deferribacterales	Deferribacteraceae	Mucispirillum	0,116	0,061	0,000	0,017	0,048	0,129	0,000	0,000	0,000	0,342	0,094	0,109	0,098	0,136	0,097	0,000	0,088	0,000	0,000	0,000	0,357	0,060	0,083
Bacteria	Tenericutes	Mollicutes	Mycoplasmatales	Mycoplasmataceae	Mycoplasma	0,000	0,000	0,000	0,000	0,000	0,000	0,000	0,000	0,000	0,000	0,000	0,059	0,000	0,148	0,000	0,000	0,041	0,040	0,043	0,000	0,000	0,000	0,017
Bacteria	Bacteroidetes	Flavobacteriia	Flavobacteriales	Flavobacteriaceae	Myroides	0,000	0,000	0,000	0,000	0,000	0,000	0,000	0,000	0,000	0,000	0,000	0,000	0,017	0,000	0,000	0,000	0,003	0,000	0,000	0,000	0,000	0,000	0,000
Bacteria	Bacteroidetes	Bacteroidia	Bacteroidales	[Odoribacteraceae]	Odoribacter	0,000	0,000	0,000	0,000	0,000	0,000	0,000	0,000	0,000	0,000	0,000	0,000	0,000	0,000	0,000	0,000	0,000	0,000	0,000	0,000	0,000	0,000	0,000
Bacteria	Actinobacteria	Coriobacteriia	Coriobacteriales	Coriobacteriaceae	Olsenella	0,000	0,000	0,000	0,000	0,000	0,000	0,000	0,000	0,000	0,000	0,000	0,000	0,000	0,000	0,000	0,000	0,000	0,000	0,000	0,000	0,000	0,000	0,000
Bacteria	Firmicutes	Clostridia	Clostridiales	Ruminococcaceae	Oscillospira	0,000	0,000	0,000	0,000	0,000	0,000	0,000	0,098	0,000	0,000	0,020	0,000	0,054	0,000	0,000	0,000	0,011	0,000	0,000	0,000	0,000	0,000	0,000
Bacteria	Firmicutes	Clostridia	Clostridiales	Ruminococcaceae	Papillibacter	0,231	0,159	0,313	0,194	0,224	0,125	0,281	0,134	0,057	0,230	0,166	0,403	0,072	0,283	0,097	0,262	0,224	0,882	0,339	0,215	0,533	0,108	0,415
Bacteria	Bacteroidetes	Bacteroidia	Bacteroidales	Porphyromonadaceae	Parabacteroides	0,000	0,000	0,000	0,000	0,000	0,000	0,000	0,000	0,000	0,000	0,000	0,000	0,000	0,000	0,000	0,000	0,000	0,000	0,000	0,000	0,012	0,000	0,002
Bacteria	Bacteroidetes	Sphingobacteriia	Sphingobacteriales	Sphingobacteriaceae	Parapedobacter	0,000	0,000	0,007	0,000	0,002	0,000	0,000	0,000	0,000	0,000	0,000	0,000	0,000	0,000	0,000	0,000	0,000	0,000	0,000	0,000	0,000	0,000	0,000
Bacteria	Bacteroidetes	Bacteroidia	Bacteroidales	[Paraprevotellaceae]	Paraprevotella	0,000	0,000	0,000	0,000	0,000	0,000	0,000	0,000	0,000	0,000	0,000	0,000	0,000	0,000	0,000	0,000	0,000	0,000	0,000	0,000	0,000	0,000	0,000
Bacteria	Proteobacteria	Gammaproteobacteria	Pasteurellales	Pasteurellaceae	Pasteurella	0,000	0,000	0,000	0,000	0,000	0,000	0,000	0,000	0,000	0,000	0,000	0,000	0,000	0,000	0,000	0,000	0,000	0,000	0,000	0,000	0,000	0,000	0,000
Bacteria	Firmicutes	Bacilli	Lactobacillales	Lactobacillaceae	Pediococcus	0,000	0,000	0,000	0,000	0,000	0,000	0,000	0,000	0,000	0,000	0,000	0,000	0,000	0,000	0,000	0,000	0,000	0,000	0,000	0,000	0,000	0,000	0,000
Bacteria	Verrucomicrobia	Verrucomicrobiae	Verrucomicrobiales	Verrucomicrobiaceae	Persicirhabdus	0,000	0,000	0,000	0,000	0,000	0,000	0,000	0,000	0,000	0,000	0,000	0,000	0,000	0,000	0,000	0,000	0,000	0,000	0,000	0,000	0,000	0,000	0,000
Bacteria	Bacteroidetes	Bacteroidia	Bacteroidales	Porphyromonadaceae	Petrimonas	0,000	0,000	0,000	0,000	0,000	0,000	0,000	0,000	0,000	0,000	0,000	0,000	0,000	0,000	0,000	0,000	0,000	0,000	0,000	0,000	0,000	0,000	0,000
Bacteria	Bacteroidetes	Bacteroidia	Bacteroidales	Prevotellaceae	Prevotella	8,070	9,971	7,533	9,316	8,723	13,807	8,797	23,462	8,218	16,055	14,068	11,376	7,359	6,635	14,164	10,047	9,916	16,916	9,363	8,606	5,574	8,792	9,850
Bacteria	Proteobacteria	Gammaproteobacteria	Enterobacteriales	Enterobacteriaceae	Pseudomonas	0,000	0,000	0,000	0,000	0,000	0,000	0,000	0,000	0,000	0,000	0,000	0,000	0,000	0,000	0,000	0,000	0,000	0,000	0,000	0,000	0,000	0,000	0,000
Bacteria	Proteobacteria	Gammaproteobacteria	Xanthomonadales	Xanthomonadaceae	Pseudoxanthomonas	0,000	0,000	0,000	0,000	0,000	0,000	0,000	0,000	0,000	0,000	0,000	0,000	0,000	0,000	0,000	0,000	0,000	0,000	0,000	0,000	0,000	0,000	0,000
Bacteria	Proteobacteria	Gammaproteobacteria	Pseudomonadales	Moraxellaceae	Psychrobacter	0,000	0,000	0,000	0,000	0,000	0,000	0,000	0,000	0,036	0,000	0,007	0,000	0,009	0,000	0,000	0,000	0,002	0,000	0,000	0,000	0,000	0,000	0,000
Bacteria	Synergistetes	Synergistia	Synergistales	Dethiosulfovibrionaceae	Pyramidobacter	0,000	0,000	0,000	0,000	0,000	0,000	0,000	0,000	0,000	0,000	0,000	0,000	0,000	0,000	0,000	0,000	0,000	0,000	0,000	0,000	0,000	0,000	0,000
Bacteria	Firmicutes	Erysipelotrichi	Erysipelotrichales	Erysipelotrichaceae	RFN20	0,000	0,000	0,000	0,000	0,000	0,000	0,007	0,000	0,000	0,000	0,001	0,000	0,000	0,000	0,000	0,000	0,000	0,000	0,000	0,000	0,000	0,000	0,000
Bacteria	Proteobacteria	Alphaproteobacteria	Rhizobiales	Rhizobiaceae	Rhizobium	0,000	0,000	0,000	0,000	0,000	0,000	0,000	0,000	0,000	0,000	0,000	0,000	0,000	0,000	0,000	0,000	0,000	0,000	0,000	0,000	0,000	0,000	0,000
Bacteria	Firmicutes	Clostridia	Clostridiales	Lachnospiraceae	Roseburia	0,462	0,930	0,000	0,713	0,526	0,716	0,000	0,456	0,312	1,049	0,507	0,126	0,933	0,333	0,354	0,950	0,539	0,000	0,637	1,177	0,000	0,306	0,424
Bacteria	Actinobacteria	Actinobacteria	Actinomycetales	Micrococcaceae	Rothia	0,000	0,037	0,000	0,000	0,009	0,000	0,000	0,000	0,000	0,000	0,000	0,000	0,000	0,000	0,000	0,000	0,000	0,000	0,000	0,000	0,000	0,000	0,000
Bacteria	Firmicutes	Clostridia	Clostridiales	Lachnospiraceae	Ruminococcus	0,111	0,067	0,000	0,000	0,044	0,000	0,000	0,000	0,000	0,120	0,024	0,507	0,321	0,304	0,120	0,000	0,251	0,000	0,146	0,000	0,000	0,198	0,069
Bacteria	Firmicutes	Clostridia	Clostridiales	Ruminococcaceae	Ruminococcus	0,000	0,000	0,209	0,000	0,052	0,000	0,098	0,000	0,000	0,000	0,020	0,193	0,225	0,031	0,000	0,000	0,090	0,302	0,267	0,000	0,000	0,123	0,138
Bacteria	Firmicutes	Clostridia	Clostridiales	Veillonellaceae	Selenomonas	1,126	1,051	0,452	2,345	1,243	1,374	2,216	1,742	0,684	2,619	1,727	1,589	0,847	0,747	0,137	1,024	0,869	2,597	1,395	0,000	1,007	1,071	1,214
Bacteria	Firmicutes	Erysipelotrichi	Erysipelotrichales	Erysipelotrichaceae	Sharpea	0,000	0,000	0,000	0,000	0,000	0,000	0,000	0,000	0,000	0,000	0,000	0,000	0,000	0,000	0,000	0,000	0,000	0,000	0,000	0,000	0,000	0,000	0,000
Bacteria	Proteobacteria	Betaproteobacteria	Burkholderiales	Comamonadaceae	Sphaerotilus	0,000	0,000	0,000	0,000	0,000	0,000	0,141	0,000	0,000	0,000	0,028	0,000	0,000	0,000	0,000	0,000	0,000	0,000	0,000	0,000	0,000	0,000	0,000
Bacteria	Spirochaetes	Spirochaetes	Spirochaetales	Spirochaetaceae	Spirochaeta	0,000	0,055	0,035	0,308	0,099	0,000	0,000	0,242	0,000	0,000	0,048	0,000	0,000	0,000	0,160	0,404	0,113	0,000	0,000	0,000	0,000	0,000	0,000
Bacteria	Firmicutes	Clostridia	Clostridiales	Ruminococcaceae	Sporobacter	19,258	6,241	5,598	4,956	9,013	6,182	44,567	11,457	3,246	3,920	13,874	1,602	9,548	11,081	4,948	3,365	6,109	2,572	7,267	14,850	5,505	2,084	6,456
Bacteria	Firmicutes	Bacilli	Bacillales	Planococcaceae	Staphylococcus	0,000	0,000	0,000	0,000	0,000	0,000	0,000	0,000	0,000	0,000	0,000	0,000	0,000	0,000	0,000	0,042	0,008	0,000	0,000	0,000	0,000	0,000	0,000
Bacteria	Firmicutes	Bacilli	Lactobacillales	Streptococcaceae	Streptococcus	0,258	0,000	0,000	0,063	0,080	0,148	0,082	0,214	0,049	0,175	0,134	0,000	0,143	0,040	0,000	0,000	0,037	0,000	0,149	0,000	0,215	0,035	0,080
Bacteria	Firmicutes	Clostridia	Clostridiales	Ruminococcaceae	Subdoligranulum	0,000	0,000	0,000	0,000	0,000	0,420	0,134	0,000	0,000	0,175	0,146	0,000	0,000	0,000	0,000	0,000	0,000	0,000	0,000	0,000	0,000	0,000	0,000
Bacteria	Firmicutes	Clostridia	Clostridiales	Veillonellaceae	Succinispira	0,360	0,640	0,547	0,333	0,470	0,083	0,219	0,000	0,233	0,233	0,154	0,164	0,676	0,398	0,578	0,000	0,363	0,324	0,166	0,000	0,000	0,123	0,123
Bacteria	Bacteroidetes	[Saprospirae]	[Saprospirales]	Chitinophagaceae	Trachelomonas	0,000	0,000	0,024	0,000	0,006	0,000	0,000	0,000	0,000	0,000	0,000	0,000	0,000	0,000	0,000	0,000	0,000	0,000	0,000	0,000	0,000	0,000	0,000
Bacteria	Spirochaetes	Spirochaetes	Spirochaetales	Spirochaetaceae	Treponema	0,589	0,115	0,789	0,641	0,533	0,000	0,000	0,043	0,000	0,066	0,022	0,176	0,253	0,163	0,328	0,168	0,217	0,028	0,034	0,000	0,000	0,137	0,040
Bacteria	Firmicutes	Bacilli	Turicibacterales	Turicibacteraceae	Turicibacter	0,000	0,000	0,000	0,000	0,000	0,000	0,000	0,000	0,000	0,000	0,000	0,000	0,000	0,000	0,000	0,000	0,000	0,000	0,000	0,000	0,000	0,000	0,000
Bacteria	Verrucomicrobia	Opitutae	[Cerasicoccales]	[Cerasicoccaceae]	Unclassified	0,000	0,000	0,000	0,000	0,000	0,000	0,000	0,000	0,000	0,000	0,000	0,000	0,000	0,000	0,000	0,000	0,000	0,000	0,000	0,000	0,000	0,000	0,000
Bacteria	Bacteroidetes	[Saprospirae]	[Saprospirales]	Chitinophagaceae	Unclassified	0,000	0,000	0,000	0,000	0,000	0,000	0,000	0,000	0,000	0,000	0,000	0,000	0,000	0,012	0,000	0,000	0,002	0,000	0,000	0,000	0,000	0,000	0,000
Bacteria	Tenericutes	Mollicutes	Acholeplasmatales	Acholeplasmataceae	Unclassified	0,000	0,000	0,000	0,000	0,000	0,000	0,000	0,000	0,000	0,000	0,000	0,000	0,000	0,000	0,000	0,000	0,000	0,000	0,000	0,000	0,000	0,000	0,000
Bacteria	Tenericutes	Mollicutes	Acholeplasmatales	Unclassified	Unclassified	0,000	0,000	0,000	0,000	0,000	0,000	0,000	0,000	0,000	0,051	0,010	0,000	0,000	0,000	0,000	0,000	0,000	0,000	0,000	0,000	0,000	0,000	0,000
Bacteria	Tenericutes	Mollicutes	Acholeplasmatales	Unclassified	Unclassified	0,000	0,000	0,000	0,810	0,202	0,000	0,000	0,000	0,000	0,000	0,000	0,000	0,000	0,000	0,000	0,000	0,000	0,000	0,000	0,000	0,000	0,000	0,000
Bacteria	Actinobacteria	Actinobacteria	Actinomycetales	Dietziaceae	Unclassified	0,000	0,000	0,000	0,000	0,000	0,000	0,000	0,000	0,000	0,000	0,000	0,059	0,000	0,000	0,000	0,000	0,012	0,000	0,000	0,000	0,000	0,000	0,000
Bacteria	Actinobacteria	Actinobacteria	Actinomycetales	Microbacteriaceae	Unclassified	0,000	0,000	0,000	0,000	0,000	0,000	0,000	0,000	0,000	0,000	0,000	0,000	0,000	0,000	0,037	0,000	0,007	0,000	0,000	0,000	0,000	0,000	0,000
Bacteria	Actinobacteria	Actinobacteria	Actinomycetales	Propionibacteriaceae	Unclassified	0,000	0,000	0,000	0,000	0,000	0,000	0,000	0,000	0,000	0,000	0,000	0,059	0,000	0,000	0,020	0,037	0,023	0,000	0,000	0,000	0,000	0,000	0,000
Bacteria	Actinobacteria	Actinobacteria	Actinomycetales	Unclassified	Unclassified	0,000	0,112	0,220	0,169	0,125	0,000	0,000	0,217	0,000	0,204	0,084	0,000	0,000	0,179	0,000	0,000	0,036	0,000	0,000	0,000	0,000	0,103	0,021
Bacteria	Proteobacteria	Gammaproteobacteria	Aeromonadales	Succinivibrionaceae	Unclassified	0,000	0,000	0,000	0,000	0,000	0,000	0,000	0,000	0,000	0,244	0,049	0,000	0,000	0,000	0,000	0,000	0,000	0,000	0,000	0,000	0,000	0,000	0,000
Bacteria	Tenericutes	Mollicutes	Anaeroplasmatales	Anaeroplasmataceae	Unclassified	0,000	0,000	0,000	0,000	0,000	0,000	0,000	0,000	0,000	0,000	0,000	0,008	0,000	0,000	0,000	0,000	0,002	0,000	0,000	0,000	0,000	0,000	0,000
Bacteria	Tenericutes	Mollicutes	Anaeroplasmatales	Anaeroplasmataceae	Unclassified	0,000	0,000	0,000	0,076	0,019	0,000	0,000	0,000	0,000	0,015	0,003	0,000	0,009	0,031	0,000	0,000	0,008	0,000	0,000	0,000	0,000	0,000	0,000
Bacteria	Chloroflexi	TK10	B07_WMSP1	FFCH4570	Unclassified	0,000	0,000	0,000	0,000	0,000	0,000	0,000	0,000	0,000	0,000	0,000	0,000	0,000	0,000	0,000	0,016	0,003	0,000	0,000	0,000	0,000	0,000	0,000
Bacteria	Firmicutes	Bacilli	Bacillales	Bacillaceae	Unclassified	0,485	1,180	0,044	0,304	0,503	0,000	0,000	0,000	0,000	0,441	0,088	1,061	0,368	0,291	0,415	0,656	0,558	0,362	0,445	0,000	0,082	0,224	0,223
Bacteria	Firmicutes	Bacilli	Bacillales	Unclassified	Unclassified	0,439	0,526	0,233	0,000	0,300	0,000	0,638	0,442	0,430	0,000	0,302	0,000	0,242	0,000	0,127	0,000	0,074	0,699	0,330	0,000	0,342	0,327	0,340
Bacteria	Bacteroidetes	Bacteroidia	Bacteroidales	[Paraprevotellaceae]	Unclassified	0,369	0,184	0,185	0,574	0,328	0,337	0,484	0,196	0,134	0,452	0,321	0,323	0,370	0,000	1,040	1,013	0,549	0,523	0,376	0,000	0,000	0,271	0,234
Bacteria	Bacteroidetes	Bacteroidia	Bacteroidales	[Paraprevotellaceae]	Unclassified	0,215	0,000	0,000	0,409	0,156	0,000	0,000	0,000	0,000	0,000	0,000	0,000	0,206	0,000	0,000	0,000	0,041	0,000	0,000	0,000	0,000	0,000	0,000
Bacteria	Bacteroidetes	Bacteroidia	Bacteroidales	p-2534-18B5	Unclassified	0,113	0,272	1,781	0,308	0,618	0,083	0,000	0,486	0,427	0,000	0,199	1,874	0,405	0,116	0,856	0,562	0,763	0,000	0,000	0,000	1,474	0,000	0,295
Bacteria	Bacteroidetes	Bacteroidia	Bacteroidales	Porphyromonadaceae	Unclassified	0,000	0,023	0,042	0,000	0,016	0,000	0,065	0,048	0,022	0,000	0,027	0,000	0,000	0,000	0,000	0,000	0,000	0,000	0,063	0,000	0,070	0,021	0,031
Bacteria	Bacteroidetes	Bacteroidia	Bacteroidales	Prevotellaceae	Unclassified	0,000	0,000	0,000	0,000	0,000	0,000	0,000	0,000	0,000	0,000	0,000	0,000	0,000	0,000	0,000	0,000	0,000	0,000	0,000	0,000	0,000	0,000	0,000
Bacteria	Bacteroidetes	Bacteroidia	Bacteroidales	Prevotellaceae	Unclassified	0,000	0,000	0,000	0,000	0,000	0,000	0,000	0,000	0,000	0,044	0,009	0,000	0,000	0,000	0,000	0,000	0,000	0,000	0,121	0,000	0,000	0,027	0,030
Bacteria	Bacteroidetes	Bacteroidia	Bacteroidales	RF16	Unclassified	0,000	0,004	0,000	0,000	0,001	0,000	0,000	0,000	0,000	0,000	0,000	0,000	0,000	0,000	0,000	0,000	0,000	0,000	0,000	0,000	0,000	0,000	0,000
Bacteria	Bacteroidetes	Bacteroidia	Bacteroidales	S24-7	Unclassified	0,000	0,000	0,000	0,000	0,000	0,000	0,000	0,000	0,000	0,000	0,000	0,000	0,000	0,000	0,214	0,000	0,043	0,000	0,000	0,000	0,000	0,000	0,000
Bacteria	Bacteroidetes	Bacteroidia	Bacteroidales	Unclassified	Unclassified	2,917	0,663	4,844	3,914	3,085	3,528	0,075	1,395	0,966	0,120	1,217	0,927	0,611	1,341	1,922	3,260	1,612	0,264	0,482	0,000	2,792	1,558	1,019
Bacteria	Bacteroidetes	Bacteroidia	Bacteroidales	Unclassified	Unclassified	3,477	4,094	5,910	11,952	6,358	7,049	4,657	3,875	6,064	3,738	5,077	7,707	4,410	9,578	14,987	11,333	9,603	1,830	5,158	3,676	3,387	2,761	3,362
Bacteria	TM7	TM7-3	Blgi18	Unclassified	Unclassified	0,000	0,000	0,000	0,000	0,000	0,000	0,000	0,000	0,000	0,000	0,000	0,000	0,000	0,000	0,000	0,000	0,000	0,000	0,000	0,000	0,000	0,000	0,000
Bacteria	Proteobacteria	Betaproteobacteria	Burkholderiales	Alcaligenaceae	Unclassified	0,000	0,000	0,000	0,000	0,000	0,000	0,000	0,000	0,000	0,000	0,000	0,000	0,000	0,000	0,000	0,000	0,000	0,000	0,000	0,000	0,000	0,000	0,000
Bacteria	Proteobacteria	Betaproteobacteria	Burkholderiales	Comamonadaceae	Unclassified	0,000	0,004	0,000	0,000	0,001	0,000	0,000	0,000	0,000	0,084	0,017	0,000	0,015	0,018	0,000	0,000	0,007	0,028	0,000	0,000	0,000	0,000	0,006
Bacteria	Proteobacteria	Betaproteobacteria	Burkholderiales	Oxalobacteraceae	Unclassified	0,238	0,000	0,187	0,000	0,106	0,000	0,000	0,000	0,369	0,000	0,074	0,449	0,000	0,294	0,000	0,000	0,149	0,035	0,000	0,000	0,207	0,000	0,048
Bacteria	Proteobacteria	Betaproteobacteria	Burkholderiales	Unclassified	Unclassified	0,000	0,000	0,189	0,000	0,047	0,000	0,000	0,130	0,435	0,026	0,118	0,000	0,000	0,095	0,000	0,467	0,113	0,000	0,000	0,000	0,070	0,000	0,014
Bacteria	Proteobacteria	Epsilonproteobacteria	Campylobacterales	Helicobacteraceae	Unclassified	0,000	0,000	0,000	0,000	0,000	0,000	0,000	0,000	0,000	0,000	0,000	0,000	0,000	0,000	0,000	0,000	0,000	0,000	0,000	0,000	0,000	0,000	0,000
Bacteria	Proteobacteria	Alphaproteobacteria	Caulobacterales	Caulobacteraceae	Unclassified	0,000	0,000	0,000	0,000	0,000	0,000	0,000	0,000	0,000	0,000	0,000	0,000	0,000	0,000	0,000	0,000	0,000	0,000	0,000	0,000	0,000	0,000	0,000
Bacteria	Chlamydiae	Chlamydiia	Chlamydiales	Unclassified	Unclassified	0,000	0,000	0,000	0,000	0,000	0,000	0,000	0,000	0,000	0,000	0,000	0,000	0,000	0,000	0,000	0,000	0,000	0,000	0,000	0,000	0,000	0,015	0,003
Bacteria	Firmicutes	Clostridia	Clostridiales	Lachnospiraceae	Unclassified	0,892	1,094	1,759	1,379	1,281	0,988	0,697	1,958	1,095	2,448	1,437	0,344	2,154	1,559	1,926	1,165	1,430	1,111	1,504	0,438	1,933	0,881	1,173
Bacteria	Firmicutes	Clostridia	Clostridiales	Peptostreptococcaceae	Unclassified	0,401	2,687	0,601	0,717	1,102	1,564	0,589	1,468	0,085	1,220	0,985	6,441	1,347	2,068	1,969	1,071	2,579	1,463	1,352	2,233	0,402	0,552	1,200
Bacteria	Firmicutes	Clostridia	Clostridiales	Ruminococcaceae	Unclassified	9,073	8,566	8,280	11,564	9,371	9,779	8,568	7,008	10,120	8,590	8,813	6,789	9,470	8,394	10,644	12,042	9,468	13,398	9,460	9,851	12,450	11,166	11,265
Bacteria	Firmicutes	Clostridia	Clostridiales	Ruminococcaceae	Unclassified	3,590	4,446	6,099	3,429	4,391	4,759	3,921	3,588	3,960	5,104	4,266	5,468	3,858	3,730	5,068	4,961	4,617	4,581	7,462	1,804	3,551	5,214	4,522
Bacteria	Firmicutes	Clostridia	Clostridiales	Veillonellaceae	Unclassified	0,528	0,317	0,291	0,148	0,321	0,170	0,059	0,408	0,000	0,313	0,190	0,281	0,749	0,075	0,164	0,136	0,281	0,088	0,000	0,000	0,000	0,558	0,129
Bacteria	Firmicutes	Clostridia	Clostridiales	Unclassified	Unclassified	6,992	9,502	10,509	7,684	8,672	10,683	1,476	11,592	10,736	8,029	8,503	6,009	11,666	9,651	3,711	9,081	8,024	4,875	7,345	2,010	8,334	9,647	6,442
Bacteria	Firmicutes	Clostridia	Clostridiales	Unclassified	Unclassified	3,914	3,441	0,327	1,666	2,337	4,846	0,350	2,788	2,403	4,189	2,915	0,721	3,131	2,174	2,233	1,953	2,042	3,105	3,427	1,331	2,956	1,653	2,495
Bacteria	Actinobacteria	Coriobacteriia	Coriobacteriales	Coriobacteriaceae	Unclassified	0,000	0,000	0,000	0,000	0,000	0,000	0,000	0,000	0,000	0,000	0,000	0,000	0,000	0,000	0,000	0,000	0,000	0,000	0,000	0,000	0,000	0,000	0,000
Bacteria	TM7	TM7-3	CW040	F16	Unclassified	0,231	0,646	0,603	0,789	0,567	0,000	0,000	0,000	0,000	0,761	0,152	0,138	0,983	0,315	0,528	0,667	0,526	0,644	0,373	0,000	0,310	0,221	0,309
Bacteria	TM7	TM7-3	CW040	Unclassified	Unclassified	0,000	0,000	0,000	0,000	0,000	0,000	0,000	0,000	0,000	0,000	0,000	0,000	0,000	0,000	0,000	0,000	0,000	0,000	0,000	0,000	0,000	0,000	0,000
Bacteria	Elusimicrobia	Elusimicrobia	Elusimicrobiales	Elusimicrobiaceae	Unclassified	0,000	0,000	0,000	0,000	0,000	0,000	0,000	0,000	0,000	0,000	0,000	0,000	0,000	0,000	0,000	0,000	0,000	0,000	0,000	0,000	0,000	0,000	0,000
Bacteria	Proteobacteria	Gammaproteobacteria	Enterobacteriales	Enterobacteriaceae	Unclassified	0,000	0,000	0,000	0,000	0,000	0,000	0,000	0,057	0,000	0,321	0,076	0,423	0,173	0,230	0,284	0,000	0,222	0,000	0,000	0,000	0,000	0,000	0,000
Bacteria	Tenericutes	Mollicutes	Entomoplasmatales	Entomoplasmataceae	Unclassified	0,000	0,000	0,000	0,000	0,000	0,000	0,000	0,000	0,000	0,000	0,000	0,000	0,000	0,000	0,000	0,000	0,000	0,000	0,000	0,000	0,000	0,000	0,000
Bacteria	Firmicutes	Erysipelotrichi	Erysipelotrichales	Erysipelotrichaceae	Unclassified	4,634	2,863	2,447	2,918	3,216	2,324	1,931	3,018	3,426	4,455	3,031	3,841	5,263	5,754	1,140	2,672	3,734	3,050	8,358	24,315	2,331	8,020	9,215
Bacteria	Bacteroidetes	Flavobacteriia	Flavobacteriales	Flavobacteriaceae	Unclassified	0,000	0,000	0,000	0,013	0,003	0,000	0,000	0,000	0,000	0,000	0,000	0,008	0,000	0,000	0,000	0,000	0,002	0,000	0,000	0,000	0,000	0,000	0,000
Bacteria	Fusobacteria	Fusobacteriia	Fusobacteriales	Fusobacteriaceae	Unclassified	0,000	0,000	0,000	0,000	0,000	0,000	0,000	0,000	0,000	0,000	0,000	0,000	0,000	0,000	0,000	0,000	0,000	0,000	0,000	0,000	0,000	0,000	0,000
Bacteria	Firmicutes	Bacilli	Lactobacillales	Lactobacillaceae	Unclassified	0,000	0,020	0,069	0,000	0,022	0,000	0,000	0,255	0,000	0,000	0,051	0,092	0,000	0,000	0,000	0,000	0,018	0,126	0,000	0,000	0,000	0,182	0,062
Bacteria	Firmicutes	Bacilli	Lactobacillales	Unclassified	Unclassified	0,000	0,000	0,000	0,000	0,000	0,000	0,000	0,000	0,003	0,000	0,001	0,000	0,000	0,000	0,000	0,000	0,000	0,000	0,000	0,000	0,000	0,000	0,000
Bacteria	Proteobacteria	Betaproteobacteria	Neisseriales	Neisseriaceae	Unclassified	0,000	0,000	0,000	0,000	0,000	0,000	0,000	0,000	0,000	0,000	0,000	0,000	0,000	0,000	0,020	0,000	0,004	0,000	0,000	0,000	0,000	0,000	0,000
Bacteria	Cyanobacteria	Oscillatoriophycideae	Oscillatoriales	Phormidiaceae	Unclassified	0,000	0,000	0,000	0,000	0,000	0,000	0,000	0,000	0,000	0,000	0,000	0,000	0,000	0,000	0,000	0,000	0,000	0,000	0,000	0,000	0,000	0,000	0,000
Bacteria	Proteobacteria	Gammaproteobacteria	Pasteurellales	Pasteurellaceae	Unclassified	0,000	0,000	0,000	0,000	0,000	0,000	0,000	0,000	0,000	0,000	0,000	0,000	0,000	0,000	0,000	0,000	0,000	0,000	0,000	0,000	0,000	0,000	0,000
Bacteria	Planctomycetes	Planctomycetia	Pirellulales	Pirellulaceae	Unclassified	0,000	0,000	0,000	0,000	0,000	0,000	0,000	0,000	0,000	0,000	0,000	0,000	0,000	0,000	0,000	0,000	0,000	0,000	0,000	0,000	0,000	0,000	0,000
Bacteria	Proteobacteria	Gammaproteobacteria	Pseudomonadales	Moraxellaceae	Unclassified	0,000	0,084	0,000	0,000	0,021	0,110	0,000	0,091	0,000	0,000	0,040	0,000	0,000	0,178	0,000	0,000	0,036	0,146	0,255	0,000	0,051	0,000	0,091
Bacteria	Proteobacteria	Alphaproteobacteria	RF32	Unclassified	Unclassified	0,000	0,000	0,000	0,000	0,000	0,000	0,000	0,000	0,000	0,000	0,000	0,000	0,000	0,000	0,000	0,000	0,000	0,000	0,000	0,000	0,000	0,000	0,000
Bacteria	Proteobacteria	Alphaproteobacteria	Rhizobiales	Unclassified	Unclassified	0,000	0,000	0,000	0,008	0,002	0,000	0,000	0,000	0,000	0,000	0,000	0,000	0,000	0,000	0,000	0,000	0,000	0,000	0,000	0,000	0,000	0,000	0,000
Bacteria	Proteobacteria	Betaproteobacteria	Rhodocyclales	Rhodocyclaceae	Unclassified	0,000	0,000	0,000	0,000	0,000	0,000	0,000	0,000	0,016	0,051	0,013	0,000	0,000	0,000	0,000	0,000	0,000	0,000	0,000	0,000	0,000	0,000	0,000
Bacteria	Proteobacteria	Alphaproteobacteria	Rhodospirillales	Acetobacteraceae	Unclassified	0,000	0,000	0,000	0,000	0,000	0,000	0,000	0,000	0,019	0,000	0,004	0,000	0,000	0,000	0,000	0,000	0,000	0,000	0,000	0,000	0,000	0,000	0,000
Bacteria	Proteobacteria	Alphaproteobacteria	Rhodospirillales	Rhodospirillaceae	Unclassified	0,000	0,000	0,581	0,000	0,145	0,000	0,000	0,000	0,000	0,000	0,000	0,168	0,000	0,000	0,000	0,000	0,034	0,000	0,000	0,000	0,849	0,000	0,170
Bacteria	Proteobacteria	Alphaproteobacteria	Rhodospirillales	Unclassified	Unclassified	0,226	0,147	0,901	0,207	0,370	0,000	0,000	0,000	0,000	0,000	0,000	0,507	0,000	0,118	0,000	0,000	0,125	0,000	0,000	0,000	1,279	0,185	0,293
Bacteria	Bacteroidetes	Sphingobacteriia	Sphingobacteriales	Sphingobacteriaceae	Unclassified	0,000	0,000	0,004	0,000	0,001	0,000	0,000	0,000	0,000	0,000	0,000	0,000	0,000	0,000	0,000	0,000	0,000	0,000	0,000	0,000	0,000	0,000	0,000
Bacteria	Proteobacteria	Alphaproteobacteria	Sphingomonadales	Sphingomonadaceae	Unclassified	0,000	0,000	0,000	0,000	0,000	0,000	0,000	0,000	0,000	0,000	0,000	0,000	0,000	0,000	0,000	0,000	0,000	0,000	0,000	0,000	0,000	0,000	0,000
Bacteria	Spirochaetes	Spirochaetes	Spirochaetales	Spirochaetaceae	Unclassified	0,127	0,102	0,022	0,051	0,075	0,000	0,000	0,000	0,016	0,000	0,003	0,616	0,193	0,000	0,211	0,336	0,271	0,000	0,000	0,000	0,119	0,132	0,050
Bacteria	Synergistetes	Synergistia	Synergistales	Synergistaceae	Unclassified	0,000	0,000	0,000	0,000	0,000	0,000	0,000	0,000	0,000	0,000	0,000	0,000	0,000	0,000	0,000	0,000	0,000	0,000	0,000	0,000	0,000	0,000	0,000
Bacteria	Synergistetes	Synergistia	Synergistales	Synergistaceae	Unclassified	0,000	0,000	0,000	0,000	0,000	0,000	0,000	0,000	0,000	0,000	0,000	0,000	0,000	0,000	0,000	0,000	0,000	0,000	0,000	0,000	0,000	0,000	0,000
Bacteria	Synergistetes	Synergistia	Synergistales	Unclassified	Unclassified	0,000	0,000	0,000	0,000	0,000	0,000	0,000	0,000	0,000	0,000	0,000	0,000	0,000	0,000	0,000	0,000	0,000	0,000	0,000	0,000	0,000	0,000	0,000
Bacteria	Proteobacteria	Betaproteobacteria	Tremblayales	Unclassified	Unclassified	0,000	0,000	0,000	0,000	0,000	0,000	0,000	0,000	0,000	0,000	0,000	0,000	0,000	0,000	0,000	0,000	0,000	0,000	0,000	0,000	0,170	0,000	0,034
Bacteria	Verrucomicrobia	Verrucomicrobiae	Verrucomicrobiales	Verrucomicrobiaceae	Unclassified	0,000	0,000	0,000	0,000	0,000	0,000	0,000	0,000	0,000	0,000	0,000	0,000	0,000	0,000	0,000	0,000	0,000	0,000	0,000	0,000	0,000	0,034	0,007
Bacteria	Lentisphaerae	[Lentisphaeria]	Victivallales	Victivallaceae	Unclassified	0,000	0,000	0,000	0,000	0,000	1,049	0,000	0,253	0,213	0,120	0,327	0,084	0,000	0,033	0,000	0,110	0,045	0,000	0,000	0,000	0,000	0,000	0,000
Bacteria	Lentisphaerae	[Lentisphaeria]	Victivallales	Victivallaceae	Unclassified	0,000	0,000	0,000	0,000	0,000	0,000	0,000	0,000	0,000	0,000	0,000	0,000	0,000	0,000	0,000	0,000	0,000	0,000	0,356	0,000	0,000	0,000	0,071
Bacteria	Verrucomicrobia	Verruco-5	WCHB1-41	RFP12	Unclassified	0,181	0,215	0,307	0,236	0,235	0,000	0,000	0,000	0,000	0,000	0,000	0,692	0,795	0,336	0,000	0,000	0,365	0,000	0,000	0,000	0,000	0,000	0,000
Bacteria	Verrucomicrobia	Verruco-5	WCHB1-41	WCHB1-25	Unclassified	0,000	0,000	0,035	0,000	0,009	0,000	0,000	0,000	0,000	0,000	0,000	0,000	0,000	0,000	0,000	0,000	0,000	0,000	0,000	0,000	0,000	0,000	0,000
Bacteria	Cyanobacteria	4C0d-2	YS2	Unclassified	Unclassified	0,147	0,530	0,191	0,198	0,267	0,000	0,000	0,417	0,000	0,000	0,083	0,021	0,033	0,100	0,000	0,100	0,051	0,000	0,000	0,000	0,732	0,063	0,159
Bacteria	Unclassified	Unclassified	Unclassified	Unclassified	Unclassified	7,698	7,910	13,470	13,466	10,636	10,774	0,772	4,938	28,965	10,226	11,135	11,497	8,375	8,153	5,770	8,934	8,546	12,222	7,818	11,492	22,112	25,472	15,823
Bacteria	Bacteroidetes	Unclassified	Unclassified	Unclassified	Unclassified	1,653	1,440	3,054	1,037	1,796	0,481	0,007	0,559	0,848	0,255	0,430	2,092	1,005	1,277	3,397	0,945	1,743	0,000	0,425	0,000	2,431	0,477	0,667
Bacteria	Firmicutes	Unclassified	Unclassified	Unclassified	Unclassified	3,008	2,785	3,830	2,590	3,053	2,555	0,959	2,229	3,071	2,911	2,345	3,170	3,365	2,123	1,638	2,892	2,638	3,075	2,735	1,202	2,928	2,612	2,511
Bacteria	Spirochaetes	Spirochaetes	Unclassified	Unclassified	Unclassified	0,904	0,341	0,601	1,029	0,719	0,541	0,000	0,228	0,348	0,000	0,223	1,459	1,200	1,350	1,237	1,927	1,435	0,000	0,023	0,000	0,000	0,434	0,091
Bacteria	Firmicutes	Clostridia	Unclassified	Unclassified	Unclassified	0,317	0,845	0,890	1,143	0,799	1,193	0,262	0,235	0,033	0,164	0,377	0,981	0,368	0,746	0,441	1,207	0,749	0,000	1,085	1,013	0,275	0,343	0,543
Bacteria	Unclassified	Unclassified	Unclassified	Unclassified	Unclassified	0,152	0,110	0,420	0,333	0,254	0,000	0,000	0,210	0,057	0,295	0,112	0,021	0,544	0,136	0,154	0,000	0,171	0,181	0,772	0,000	0,139	0,077	0,234
Bacteria	Proteobacteria	Alphaproteobacteria	Unclassified	Unclassified	Unclassified	0,000	0,000	0,000	0,000	0,000	0,000	0,000	0,000	0,961	0,000	0,192	1,484	0,052	0,000	0,140	0,000	0,335	0,035	0,066	0,000	0,771	0,276	0,230
Bacteria	Proteobacteria	Unclassified	Unclassified	Unclassified	Unclassified	0,362	0,202	0,283	0,388	0,309	0,000	0,000	0,000	0,033	0,000	0,007	0,050	0,000	0,000	0,000	0,000	0,010	0,000	0,000	0,000	0,209	0,000	0,042
Bacteria	Cyanobacteria	Unclassified	Unclassified	Unclassified	Unclassified	0,136	0,088	0,134	0,000	0,090	0,000	0,121	0,134	0,501	0,197	0,191	0,000	0,102	0,175	0,000	0,000	0,055	0,128	0,218	0,000	0,203	0,074	0,125
Bacteria	Tenericutes	Unclassified	Unclassified	Unclassified	Unclassified	0,000	0,000	0,000	0,000	0,000	0,000	0,000	0,000	0,000	0,000	0,000	0,000	0,000	0,000	0,000	0,000	0,000	0,000	0,000	0,000	0,000	0,000	0,000
Bacteria	Proteobacteria	Betaproteobacteria	Unclassified	Unclassified	Unclassified	0,000	0,016	0,000	0,000	0,004	0,000	0,018	0,000	0,000	0,000	0,004	0,000	0,123	0,001	0,147	0,000	0,054	0,000	0,000	0,000	0,008	0,000	0,002
Bacteria	Proteobacteria	Deltaproteobacteria	Unclassified	Unclassified	Unclassified	0,068	0,000	0,000	0,000	0,017	0,000	0,000	0,000	0,000	0,000	0,000	0,000	0,000	0,000	0,000	0,000	0,000	0,000	0,000	0,000	0,000	0,000	0,000
ssigned	Unclassified	Unclassified	Unclassified	Unclassified	Unclassified	0,000	0,000	0,000	0,000	0,000	0,000	0,000	0,000	0,000	0,000	0,000	0,000	0,000	0,000	0,000	0,000	0,000	0,000	0,000	0,000	0,000	0,000	0,000
Bacteria	Firmicutes	Bacilli	Unclassified	Unclassified	Unclassified	0,000	0,037	0,000	0,000	0,009	0,000	0,000	0,000	0,000	0,015	0,003	0,000	0,000	0,000	0,000	0,000	0,000	0,000	0,020	0,000	0,000	0,000	0,004
Bacteria	Lentisphaerae	Unclassified	Unclassified	Unclassified	Unclassified	0,159	0,131	0,000	0,000	0,072	0,000	0,000	0,253	0,000	0,058	0,062	0,000	0,000	0,116	0,000	0,000	0,023	0,000	0,227	0,000	0,068	0,000	0,059
Bacteria	OD1	Unclassified	Unclassified	Unclassified	Unclassified	0,000	0,000	0,000	0,000	0,000	0,000	0,000	0,000	0,000	0,000	0,000	0,000	0,000	0,000	0,000	0,000	0,000	0,000	0,000	0,000	0,000	0,000	0,000
Bacteria	Proteobacteria	Gammaproteobacteria	Unclassified	Unclassified	Unclassified	0,163	0,000	0,000	0,000	0,041	0,000	0,000	0,000	0,000	0,000	0,000	0,000	0,000	0,000	0,000	0,000	0,000	0,000	0,347	0,000	0,000	0,244	0,118
Bacteria	Tenericutes	Mollicutes	Unclassified	Unclassified	Unclassified	0,000	0,000	0,191	0,000	0,048	0,000	0,000	0,000	0,000	0,044	0,009	1,073	0,000	0,000	0,000	0,000	0,215	0,000	0,000	0,000	0,000	0,000	0,000
Archaea	Euryarchaeota	Thermoplasmata	E2	[Methanomassiliicoccaceae]	vadinCA11	0,000	0,000	0,124	0,224	0,087	0,000	0,000	0,000	0,000	0,000	0,000	0,134	0,000	0,069	0,057	0,110	0,074	0,000	0,000	0,000	0,000	0,000	0,000
Bacteria	Bacteroidetes	Bacteroidia	Bacteroidales	Porphyromonadaceae	Vestibaculum	0,000	0,000	0,000	0,000	0,000	0,000	0,000	0,000	0,000	0,000	0,000	0,000	0,000	0,000	0,047	0,031	0,016	0,000	0,000	0,000	0,000	0,000	0,000
Bacteria	Lentisphaerae	[Lentisphaeria]	Victivallales	Victivallaceae	Victivallis	0,199	0,000	0,104	0,000	0,076	0,000	0,000	0,000	0,000	0,066	0,013	0,000	0,210	0,000	0,000	0,000	0,042	0,000	0,000	0,000	0,086	0,000	0,017
Bacteria	Spirochaetes	Spirochaetes	Sphaerochaetales	Sphaerochaetaceae	wall-less	0,000	0,000	0,000	0,000	0,000	0,000	0,000	0,000	0,000	0,000	0,000	0,000	0,000	0,000	0,000	0,000	0,000	0,000	0,000	0,000	0,000	0,000	0,000
Bacteria	Bacteroidetes	Bacteroidia	Bacteroidales	[Paraprevotellaceae]	YRC22	0,000	0,000	0,125	0,105	0,058	0,000	0,000	0,000	0,000	0,000	0,000	0,000	0,000	0,000	0,000	0,000	0,000	0,000	0,000	0,000	0,000	0,000	0,000

Supplementary Table S3. Crypt depth and villi hight in ileum and colon after FMT

Crypt depth and villi height in ileum by group (μm)

	PBS group		PF group		WF group		AWF group	
	mean	SEM	mean	SEM	mean	SEM	mean	SEM
Villi Height	344.250	17.115	334.333	10.793	336.200	8.861	334.333	12.426
Crypt Depth	217.750	7.574	221.667	12.309	223.400	7.807	224.000	10.368
V/C	1.649	0.111	1.662	0.111	1.603	0.073	1.580	0.087

V/C: Villi height/Crypt Depth ratio

Crypt depth in colon by group (μm)

	PBS group*		PF group *		WF group		AWF group	
	mean	SEM	mean	SEM	mean	SEM	mean	SEM
Crypt Depth	495	7.425	466.000	14.016	489.000	6.222	474.667	13.128

* *P* value (0.01-0.05)

Supplementary Table S4. Score for clinical signs recorded throughout the study

**Score**	**Body condition**		**Behavior**	**Digestive signs**	**Respiratory signs**	**Other signs**
	**Vertebra**	**Ribs**	**Signs**	**Signs**	**Signs**	**Dermatitis, testicular tummefaction, lameness**
0	Non visible	Non visible	Active, alert, plays	Perianal skin clean	Non apparent	Non apparent
1	Visible in the dorsum	Non visible	Sadness, quite	Perianal skin dirty	Mild dispnea	Mild
2	Prominent	Visible	Mild depression, alert	Hind limbs dirty	Evident dyspnea	Moderate
3	Very marked	Individually	Depression, stillness, postration	Red/blotchy skin lesions in the back	panting, difficult breathing	Severe

Supplementary Table S5. Core taxa list from different sampling times in PF and AWF groups and Venn diagrams representing the shared taxa.

Groups	total	family	Genera
**PF_0dpft PF_8 dpft PF_15dpft**	4	*Ruminococcaceae*	*Clostridium*
		Prevotellaceae	*Prevotella*
		Ruminococcaceae	*Sporobacter*
		Ruminococcaceae	*Papillibacter*
**PF_0dpft PF_8dpft**	1	*[Barnesiellaceae]*	*Barnesiella*
**PF_0dpft PF_15dpft**	3	*Veillonellaceae*	*Succinispira*
		Clostridiaceae	*Clostridium*
		Ruminococcaceae	*Butyricicoccus*
**PF_15dpft PF_8dpft**	2	*Erysipelotrichaceae*	*gut*
		Paraprevotellaceae	*Paraprevotella*
**PF_0dpft**	4	*Desulfovibrionaceae*	*Desulfovibrio*
		Ruminococcaceae	*Bacteroides*
		Lachnospiraceae	*Roseburia*
		Lachnospiraceae	*Ruminococcus*
**PF_8dpft**	1	*Erysipelotrichaceae*	*Anaerorhabdus*
**PF_15dpft**	7	*Spirochaetaceae*	*Treponema*
		Ruminococcaceae	*Faecalibacterium*
		Veillonellaceae	*Selenomonas*
		Pasteurellaceae	*Actinobacillus*
		Lachnospiraceae	*Clostridium*
		Campylobacteraceae	*Campylobacter*
		Erysipelotrichaceae	*Asteroleplasma*

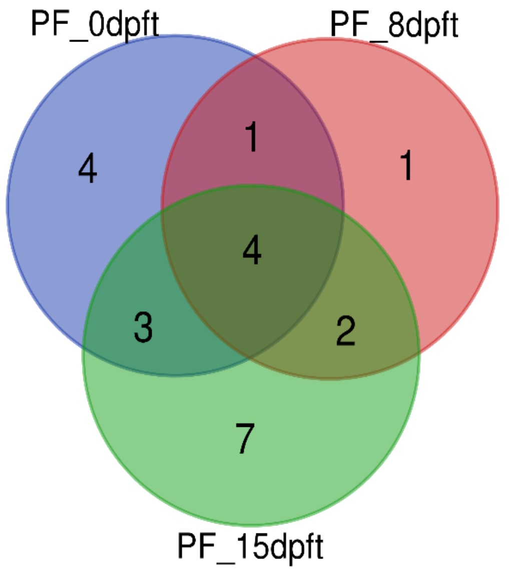

Supplementary Table S5. Core taxa list from different sampling times in PF and AWF groups and Venn diagrams representing the shared taxa (cont.).

**Groups**	**total**	**Family**	**Genera**
**AWF_0 dpft AWF_8 dpft AWF_15 dpft**	**6**	*Ruminococcaceae*	*Clostridium*
		Prevotellaceae	*Prevotella*
		[Barnesiellaceae]	*Barnesiella*
		Erysipelotrichaceae	*gut*
		Ruminococcaceae	*Sporobacter*
		Paraprevotellaceae	*Paraprevotella*
**AWF_0 dpft AWF_8 dpft**	**1**	*Veillonellaceae*	*Succinispira*
**AWF_0 dpft AWF_15 dpft**	**3**	*Erysipelotrichaceae*	*Anaerorhabdus*
		Clostridiaceae	*Clostridium*
		Ruminococcaceae	*Papillibacter*
**AWF_8 dpft AWF_15 dpft**	**2**	*Ruminococcaceae*	*Faecalibacterium*
		Erysipelotrichaceae	*Asteroleplasma*
**AWF_0dpft**	**3**	*Peptostreptococcaceae*	*Clostridium*
		Ruminococcaceae	*Butyricicoccus*
		Lachnospiraceae	*Defluviitalea*
**AWF_8dpft**	**2**	*Ruminococcaceae*	*Gemmiger*
		Lachnospiraceae	*Clostridium*
**AWF_15dpft**	**4**	*Desulfovibrionaceae*	*Desulfovibrio*
		Spirochaetaceae	*Treponema*
		Veillonellaceae	*Selenomonas*
		Lachnospiraceae	*Roseburia*

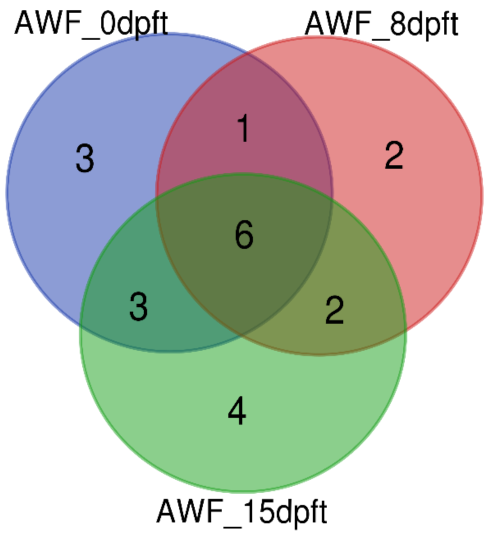

Supplementary Table S6. Average haematology parameters in pigs at 4 dpft and 15 dpft.

	4 dpft				15 dpft				Reference
	PBS	PF	WF	AWF	PBS	PF	WF	AWF	
WBC (x10E3)	21.46	22.89	24.22	23.25	24.93*	31.8	34.12*	29.8	11.0 - 22.0
RBC (x10E6)	5.93	5.79	5.92	5.64	6.45	5.99	6.17	5.84	6.8 -12.9
LYM (x10E3)	7.9	7.6	7.88	8.98	9.58	9.71	10.36	10.75	4.3 - 13.0
% LYM	43.27	40.1	37.25	39.88	40.91	33.41	34.53	37	
MONO (x10E3)	0.73	0.84	0.98	0.88	1.09	1.22	0.95	0.92	0.2 - 2.2
% MONO	3.7	3.84	4.54	3.93	4.54	3.83	3.13	3.15	
EOS (x10E3)	0.7	0.28	0.36	0.31	0.5	0.59	0.6	0.51	0.05 - 2.4
% EOS	3.88	1.38	1.48	1.34	2.06	1.93	1.78	1.76	
NEU (x10E3)	11.91	13.93	14.84	12.82	13.52*	20.06	22.00*	17.38	3.1 - 10.5
%NEU	48.14	53.48	56	53.68	51.47	60	59.85	57.26	
BASO (x10E3)	0.04	0.05	0.04	0.05	0.07	0.06	0.06	0.05	
% BASO	0.22	0.22	0.19	0.21	0.28	0.21	0.21	0.17	

**P* value < 0.05

PBS: non-transplanted group; PF: pig feces group; WF: warthog feces group; AWF: antibiotic + warthog feces group.
